# The Therapeutic Efficacy of Inhaled Vitamin D3 Metabolites Initiated During the Acute Phase of Hypersensitivity Pneumonitis: An In Vivo Study

**DOI:** 10.3390/molecules31162812

**Published:** 2026-08-12

**Authors:** Marta Kinga Lemieszek, Michał Chojnacki, Iwona Paśnik, Ilona Leśniowska, Jakub Anisiewicz, Wiktoria Gawryś, Alicja Wilczyńska, Michał Kiełbus

**Affiliations:** 1 Department of Medical Biology, Institute of Rural Health, 20-090 Lublin, Poland; chojnacki.michal@imw.lublin.pl (M.C.);; 2 Department of Biochemistry and Molecular Biology, Medical University of Lublin, 20-059 Lublin, Poland; michal.kielbus@umlub.edu.pl

**Keywords:** vitamin D, cholecalciferol, calcidiol, calcitriol, pulmonary fibrosis, hypersensitivity pneumonitis, extrinsic allergic alveolitis, *Pantoea agglomerans*

## Abstract

Our earlier studies have revealed the beneficial impact of vitamin D3 (VD3) metabolites on the development of pulmonary fibrosis in a murine model of hypersensitivity pneumonitis (HP). Despite the discovery of the great antifibrotic potential of both the bioactive form of VD3, 1,25(OH)_2_-VD3 and its direct precursor, 25(OH)-VD3, study designs based on the co-administration of VD3-metabolites and fibrosis-inducers do not reflect the clinical situation. Thus, the presented study aims to examine the effectiveness of therapeutic interventions based on the mentioned inhalations, initiated during the acute phase of HP at the first signs of pulmonary fibrosis. The research was performed using VD3-deficient mice with HP, while VD3-sufficient mice were used as the main control. The data were collected after 28 days of daily exposure of the mice to a fibrosis-inducer (an antigen of *Pantoea agglomerans*, a well-known etiological factor in HP), with 25(OH)-VD3 or 1,25(OH)_2_-VD3 administered daily during the last 14 days of the experiment. The effect of the intervention was monitored by whole-body plethysmography, histological assessment, flow cytometry, RealTime-PCR, and ELISA. Treatment with VD3 metabolites initiated during the acute phase of HP alleviated the disease courses and restored respiratory function, thanks to inhibition of pathological extracellular matrix deposition, immune-cell infiltration of lung parenchyma, profibrotic cytokine production, and EMT progression. Despite the discovery of the beneficial features of the investigated VD3-based therapies, none of them caused full recovery in mice with HP. Nevertheless, after verification in additional animal models and a deeper understanding of the underlying molecular mechanisms, the identified beneficial features of the investigated metabolites have the potential to serve as a foundation for future therapies for HP fibrosis.

## 1. Introduction

Considering an increasing incidence, low quality of life, and dramatically shortened life expectancy, pulmonary fibrosis is an important problem in medical and public health [1,2]. There are several respiratory diseases involving fibrosis, but among them, hypersensitivity pneumonitis (HP) is unique because of its well-known aetiology. HP (also known as extrinsic allergic alveolitis) represents an interstitial lung disease that occurs in genetically susceptible individuals chronically exposed to airborne antigens, which provoke hypersensitivity reactions with inflammation that may develop into pulmonary fibrosis [3,4]. HP can be caused by a diverse range of antigens, including bacteria, fungi, plant and animal proteins, as well as chemicals [3,4,5]. So far, more than 200 different antigens have been associated with the development of HP and, depending on their occurrence, different variants of this disease have been distinguished, e.g., farmer’s lung, pigeon breeder’s lung, cheese workers’ lung, mushroom worker’s lung, humidifier lung, wood fibre alveolitis, malt fever, bagassosis, or suberosis [5,6]. Because of the great variety and distribution of HP-induced antigens, millions of individuals worldwide are exposed to them as part of their occupational, home, or recreational environments, which translates into disease incidence [7,8]. Although the number of HP patients is lower than that of IPF (idiopathic pulmonary fibrosis), the weight and scale of the problem are significant enough to deserve greater interest from the scientific community, especially when we understand how insidious HP is The first acute symptoms of this disease (where inflammation dominates) are usually ignored since they usually disappear spontaneously. Failure to detect HP at this early stage promotes the gradual and silent development of pulmonary fibrosis, which is irreversible and fatal. Misdiagnoses are also a problem. Morell et al. showed that almost half of patients diagnosed with IPF, based on the American Thoracic Society and the European Respiratory Society criteria, were subsequently diagnosed with chronic HP (HP with fibrosis) [9]. Therefore, to effectively combat this disease, it is necessary to understand every single aspect of this pathology.

The results of our previous studies conducted in a well-established animal model of HP, in which pulmonary fibrosis is induced by chronic exposure of C57BL/6 mice to the antigen in *Pantoea agglomerans* (one of the key etiological factors of HP) [10,11,12,13,14,15,16], demonstrated a significant role of vitamin D3 (VD3) metabolites over the course of this disease [17,18,19]. The mentioned studies focused on cholecalciferol (a natural nutrient form of VD3), 25(OH)-VD3 (calcidiol: a faster-acting metabolised form of VD3, a direct precursor of calcitriol), and 1,25(OH)_2_-VD3 (calcitriol: a bioactive form of VD3, a hormone). The performed research, the results of which are presented in detail in the Discussion, revealed that VD3 deficiency itself (induced by a cholecalciferol-deprived diet) disturbs the immune balance and initiates other unfavourable changes in lung tissue supporting fibrosis development. Further studies proved that VD3-deficient mice had higher sensitivity compared to VD3-sufficient mice in terms of fibrosis response to an HP antigen, which was strongly associated with enhanced epithelial-to-mesenchymal transi-tion (EMT), which, in the presence of chronic inflammation in the respiratory tract, was directly responsible for pathological tissue repair and worsening respiratory functions. Moreover, fibrosis development in VD3-deficient mice was supported by deepening pulmonary calcitriol resources. A correlation between the endogenous calcitriol level and the range of pathological changes during HP development was further positively verified by the effectiveness of therapeutic intervention, based on inhalation of 25(OH)-VD3 or 1,25(OH)_2_-VD3 in amounts that restored the physiological pulmonary concentration of the bioactive form of VD3. The signs of beneficial influences of VD3 metabolites on fibrosis development over the course of HP were both an improvement of respiratory functions, as well as attenuation of inflammation and signs of fibrosis, mostly because of the silencing of the EMT phenomenon [17,18,19]. Discovering the possibility of modulating EMT by inhaled VD3 metabolites in a murine model of HP is important for at least two reasons. First of all, this is because of the biological implications of EMT. Studies over the last twenty years have shown that the basis of tissue fibrosis, including pulmonary fibrosis, is EMT [20,21,22,23,24,25]. This multidimensional phenomenon can be induced by a wide range of growth factors and cytokines. These soluble ligands interact with their specific receptors (e.g., TGFβ receptors, RTK—tyrosine kinase receptors, Frizzled, and Notch) leading to the activation of intracellular signalling pathways, including the development signalling pathways such as Wnt/β-catenin or Notch, the main inflammatory signalling pathway—NFκB, and pathways involved in growth factors like transforming growth factor β (TGFβ), fibroblast growth factor (FGF), epidermal growth factor (EGF), and insulin-like growth factor (IGF). Despite the wide range of signalling pathways that initiate EMT, they activate one or more EMT-transcription factors, such as Snail, Slug, ZEB1, and ZEB2. These factors suppress genes of epithelial markers and activate genes related to the mesenchymal phenotype [20,26,27]. Thus, discovering, in our previous studies, the inhibition of over-expressed mesenchymal cell markers (*Vim*/vimentin, *Fn1*/fibronectin, *Acta2*, and *Cdh2*) and the up-regulated EMT-involved factors (*Snail1*, *Snail2*, *Zeb1*, *Zeb2*, *NfkB*, *Tgfb*/TGFβ, and FGF2), as well as enhancement of the down-regulated epithelial cell markers (*Cdh1* and *Ocln*) in Vd3-deficent mice with HP in response to inhalation of 25(OH)-VD3 or 1,25(OH)_2_-VD3, were excellent incentives to continue this research [18,19]. An additional encouragement for the herein presented study was the unique nature of the therapeutic strategy we proposed, which involved the use of VD3 metabolites at a concentration capable of restoring physiological calcitriol levels in lung tissue, as well as delivering metabolites directly to the respiratory tract. To the best of our knowledge, our team is the first and so far the only one that has examined the possibility of using inhaled VD3 metabolites in the prevention of HP. The evaluation of the therapeutic potential of locally administered VD3 metabolites in other respiratory disorders is also very rare. There are only three studies that have investigated the influence of inhalation of vitamin D3 metabolites on the respiratory system: the improvement in the development of neonatal rat lungs in response to both 25(OH)-VD3 or 1,25(OH)_2_-VD3 [28], fighting Mycobacterium tuberculosis using 1,25(OH)_2_-VD3 [29], and the attenuation of LPS-induced acute lung inflammation also by using 1,25(OH)_2_-VD3 [30]. Moreover, alveolar regeneration in a murine model of chronic obstructive pulmonary disease (COPD) has been observed in response to both transpulmonary and intrapulmonary administration of 1,25(OH)_2_-VD3 [31,32].

Despite the great results of the above-described studies, it needs to be emphasised that data were collected from an experiment in which VD3 metabolites were administered simultaneously with a fibrosis-inducing factor (*P. agglomerans* antigen), which does not fully reflect the clinical situation. Since most HP patients seek medical attention during the acute phase of the disease, the presented studies focus on the examination of the influence of inhaled VD3 metabolites on HP-fibrosis when therapy is initiated during the indicated phase of disease development (day 14 of the experiment). The data presented herein were collected after 28 days of daily exposure of VD3-deficient mice to *P. agglomerans* antigen, with 25(OH)-VD3 or 1,25(OH)_2_-VD3 inhalation administered daily during the last 14 days of the experiment. This approach better reflects the regimen of potential therapeutic use of VD3 metabolites, bringing us closer to the development of effective antifibrotic treatment for patients with chronic HP.

## 2. Results

### 2.1. Inhaled VD3 Metabolites Reduced Disorders in Lung Tissue Morphology Caused by VD3 Deficiency and HP Development

**Figure 1:**
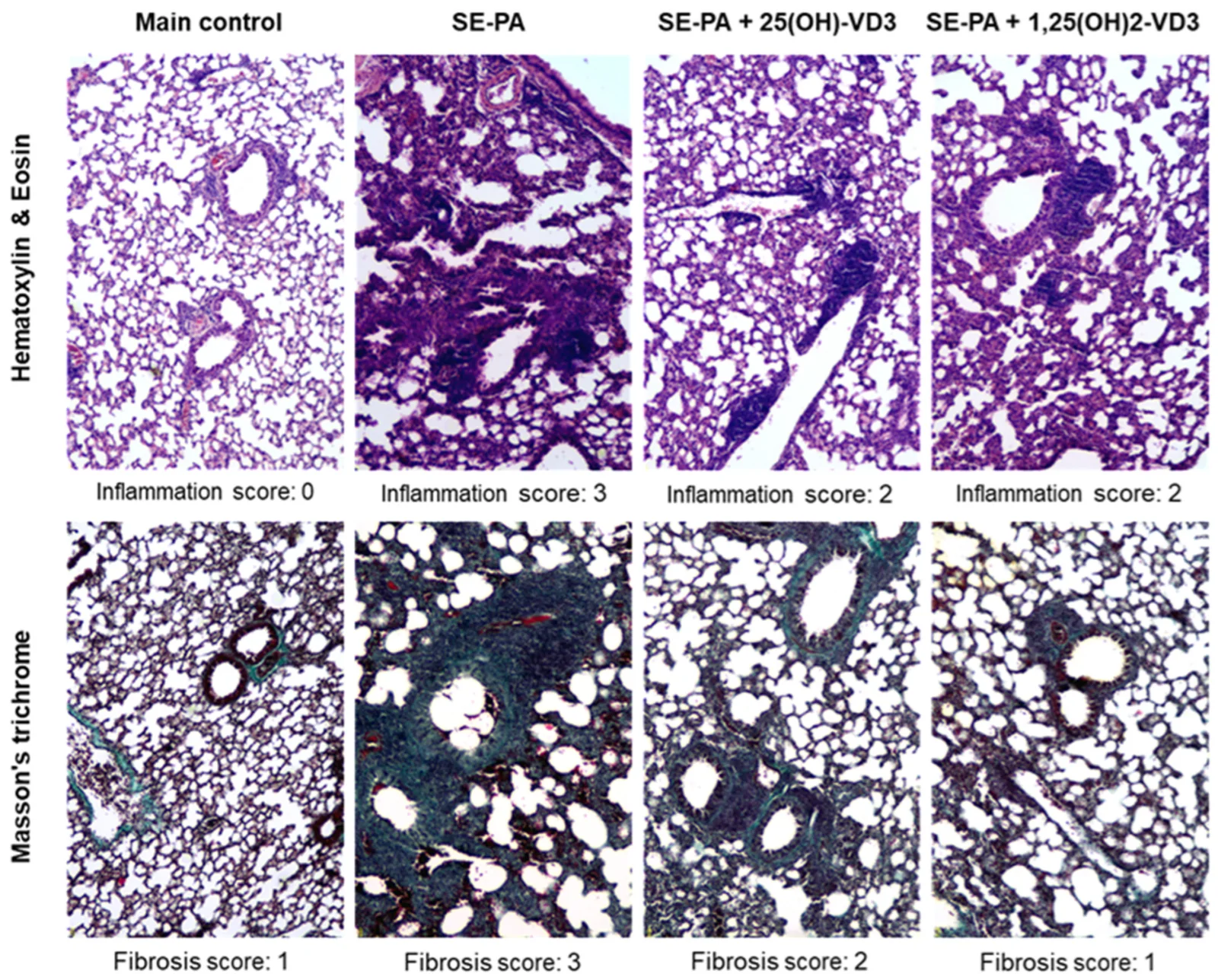
The beneficial impact of inhaled VD3 metabolites on immune response and fibrosis development in VD3-deficient mice with HP. Lung tissue collected from healthy mice (main control) as well as mice with HP with and without exposure to 25(OH)-VD3 or 1,25(OH)_2_-VD3 were subjected to Hematoxylin and Eosin, as well as Masson’s trichrome staining, and assessed under light microscopy at 100× magnification. Representative photographs of the lung sections are shown. The visual quantification of inflammation and fibrosis scores is given as the median of the investigated items. These features were graded with the 5-point Murray’s scale: 0 = regular tissue; 1 = slight injury 25%; 2 = moderate injury 50%; 3 = severe injury 75%; and 4 = very severe injury 100%.

As presented in Figure 1 and Table S1, chronic exposure of VD3-deficient mice to the *P. agglomerans* antigen triggered centrolobe and interstitial influx of immune cells (granulocytes, mostly neutrophils and reactive pneumocytes) and alterations in lung tissue morphology—the thickening of the alveolar walls leading to obstruction of the alveoli lumen. On a five-point scale (from 0 to 4), the scope of undesirable changes was assessed as a 3, from both inflammation and fibrosis. In the case of the second one, even healthy animals revealed signs of fibrosis at level 1. Negative changes caused by the antigen of *P. agglomerans* were significantly reduced by exposure of VD3-deficient mice to 25(OH)-VD3; however, the signs of inflammation and focally thickened alveolar septum were still noted (median scores for both inflammation and fibrosis decreased from 3 to 2 points). At the same time, in mice with HP, the inhalation of 1,25(OH)_2_-VD3 also limited the influx of immune cells into the lung parenchyma (median scores for inflammation were reduced from 3 to 2 points), as well as inhibited the deposition of extracellular matrix components (median scores for fibrosis decreased from 3 points to 1 point).

### 2.2. Inhaled VD3 Metabolites Relieved Respiratory Disorders Caused by HP and VD3 Deficiency

**Figure 2:**
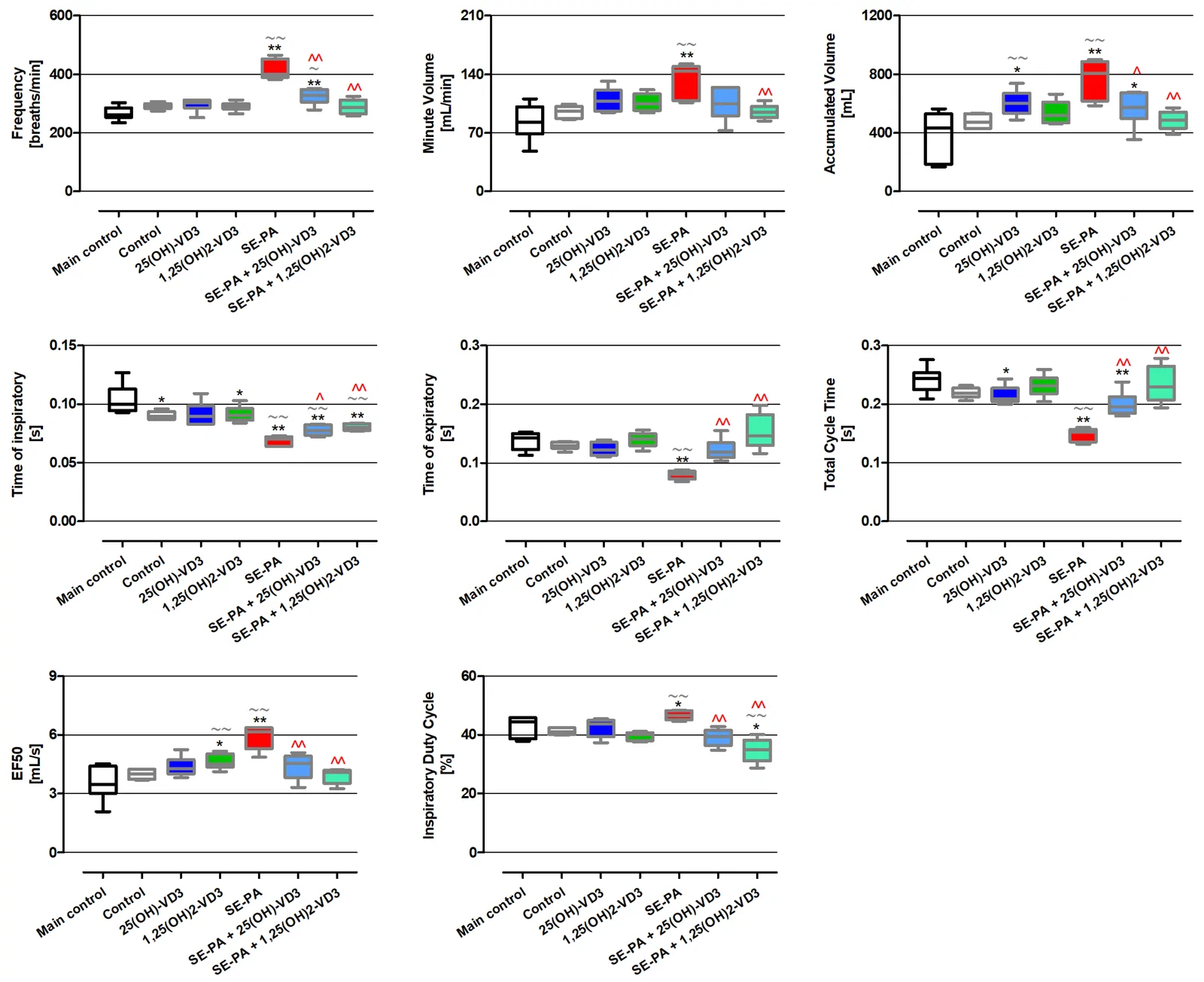
Changes in lung function observed in the VD3-deficient mice with HP treated with 25(OH)-VD3 or 1,25(OH)_2_-VD3. Respiratory parameters were examined at the end of the experiments using whole-body plethysmography. Statistical significance was examined using the Wilcoxon–Mann–Whitney test with Benjamini–Hochberg correction. Statistical comparisons are presented for: * (vs. the main control); ~ (vs. the control); and ^ (SE-PA + 25(OH)-VD3 vs. SE-PA and SE-PA + 1,25(OH)2-VD3 vs. SE-PA). The number of symbols corresponds with the significance level: one (*p* < 0.05), two (*p* < 0.01). The numerical data are listed in Supplementary Table S2.

Whole-body plethysmography (Figure 2 and Table S2) revealed significant disorders in pulmonary function in VD3-defficient mice with HP, the signs of which were increased values for the frequency of breathing (399.64 breath/min vs. 261.87 breath/min in the main control), minute volume (143.81 mL/min vs. 82.74 mL/min in the main control), accumulated volume (807.20 mL vs. 434.24 mL in the main control), EF50 (6.15 mL/s vs. 3.46 mL/s in the main control), and Inspiratory Duty Cycle (46.66% vs. 44.46% in the main control). The indicated changes were also significant compared to the control mice. Next to elevated levels of the above-mentioned respiratory parameters, the mice with HP and VD3 deficiency showed lowered values for the Total Cycle Time (0.15 s vs. 0.24 s in the main control), time of inspiration (0.07 s vs. 0.10 s in the main control), and time of expiration (0.08 vs. 0.14 s in the main control). The observed changes were also significant compared to the control mice.

Exposure of the VD3-deficient mice with developed HP to 25(OH)-VD3 beneficially impacted all investigated respiratory parameters, except MV, striving to restore the physiological level. This aim was achieved in the case of EF50, IDC, and Te, which, in response to the 25(OH)-VD3 treatment, reached the following values: 4.55 mL/s (EF50), 0.12 s (Te), and 39.45% (IDC). In the case of AV and TCT, inhalation of 25(OH)-VD3 by the mice with HP allowed reaching values observed in untreated mice on the same deficient diet (AV: 573.93 mL; TCT: 0.20 s).

The VD3-deficient mice’s inhalation of both the antigen of *P. agglomerans* and 1,25(OH)_2_-VD3 revealed even better influence than 25(OH)-VD3 on lung function. Five of seven investigated parameters disturbed by HP development returned to physiological levels in response to 1,25(OH)_2_-VD3, including F (287.06 breath/min), MV (94.79 mL/min), AV (486.44 mL), EF50 (4.08 mL/s), TCT (0.23 s), and Te (0.15 s). 1,25(OH)_2_-VD3 also significantly decreased the elevated value of Ti observed in the mice with HP; however, it was not able to restore it to the main control or control levels (0.08 s vs. 0.10 s in the main control or 0.09 s in the control). At the same time, the IDC value in the mice with HP exposed to 1,25(OH)2-VD3 was significantly below the levels in healthy animals (35% vs. 44.46% in the main control or 40.99% in the control).

### 2.3. Inhaled VD3 Metabolites Prevented Excessive Influx of Immune Cells into the Lungs of VD3-Deficient Mice with HP

**Figure 3:**
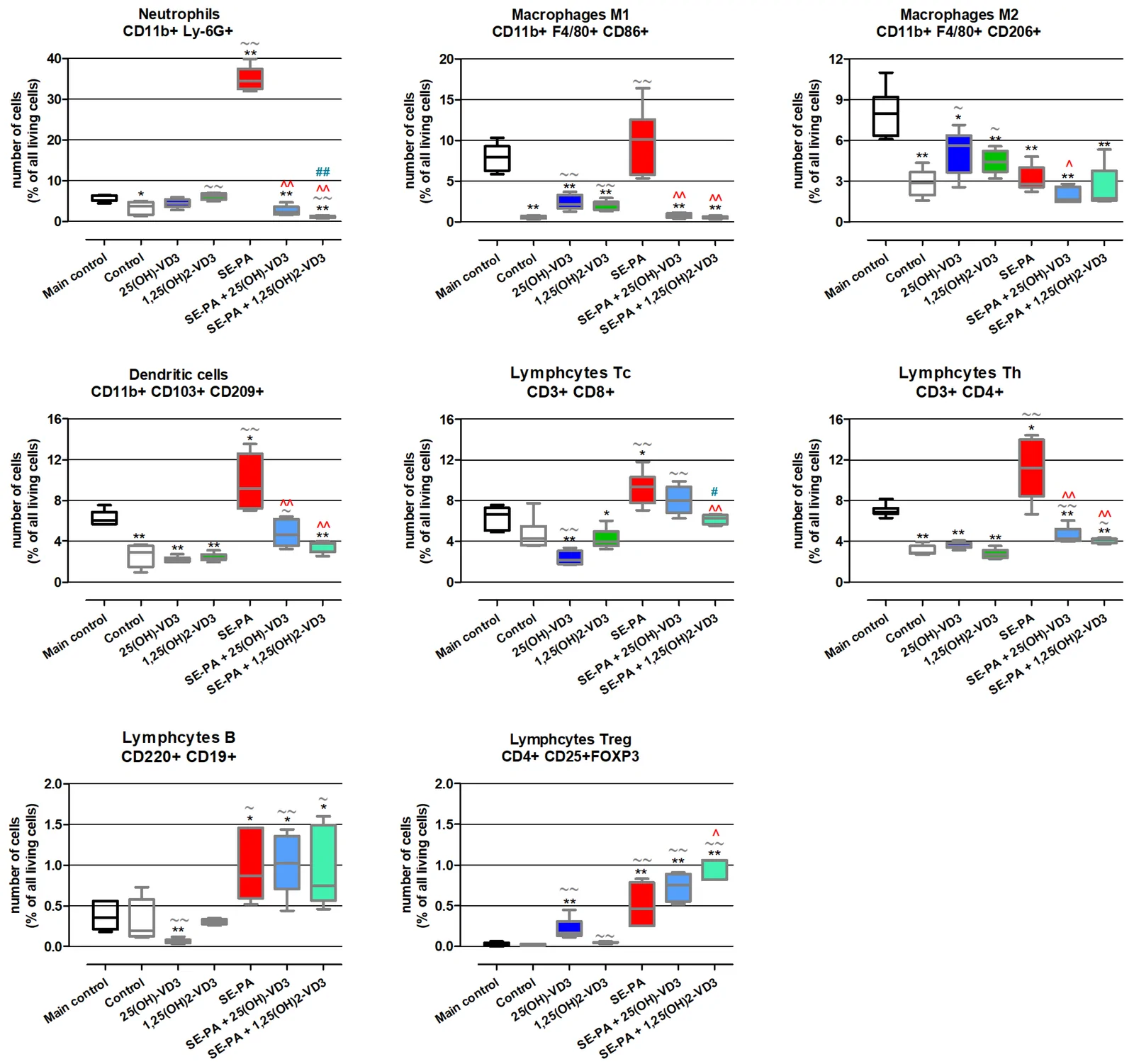
Alterations in the pulmonary composition of immune cells observed in the VD3-deficient mice with HP treated with 25(OH)-VD3 or 1,25(OH)_2_-VD3. Amounts of immune cells in lung tissue homogenates were determined using flow cytometry. Statistical significance was examined using the Wilcoxon–Mann–Whitney test with Benjamini–Hochberg correction. Statistical comparisons are presented for: * (vs. the main control); ~ (vs. the control); ^ (SE-PA + 25(OH)-VD3 vs. SE-PA and SE-PA + 1,25(OH)2-VD3 vs. SE-PA); and # (SE-PA + 25(OH)-VD3 vs. SE-PA + 1,25(OH)2-VD3). The number of symbols corresponds with the significance level: one (*p* < 0.05), two (*p* < 0.01). The numerical data are listed in Supplementary Table S3. The complete gating strategy, including the selection of the main cell population, singlet discrimination, and identification of immune cells, is presented in Supplementary Figure S3. The representative gating plots for immune cell populations across all of the experimental groups were presented in Supplementary Figure S4.

Examination of the immune cell composition of the lung homogenates collected from the VD3-defficient mice with HP (Figure 3, Figures S3 and S4, and Table S3) revealed over-accumulation of neutrophils (34.47% vs. 6.23% in the main control), lymphocytes Tc (9.36% vs. 6.64% in the main control), lymphocytes Th (11.23% vs. 6.89% in the main control), dendritic cells (9.20% vs. 6.05% in the main control), lymphocytes Treg (0.46% vs. 0.02% in the main control), and B cells (0.87% vs. 0.36% in the main control) compared to healthy animals on both the VD3-sufficient and VD3 deficient diet. On the contrary, the amount of M2 macrophages was significantly lower in the mice with HP than noted in the main control (2.79% vs. 8.01%).

The 25(OH)-VD3 treatment of the VD3-deficient mice with HP significantly inhibited the influx of neutrophils (2.29% vs. 34.47%), lymphocytes Th (4.28% vs. 11.23%) and dendritic cells (4.65% vs. 9.20%), reducing their levels below the main control. At the same time, the inhalation of 25(OH)-VD3 by the mice with HP led to a significant reduction in both M1 (10.09% vs. 0.81%) and M2 (2.79% vs. 1.64%) macrophages, and the recorded cell counts were significantly below the values observed under physiological conditions. The 25(OH)-VD3 treatment did not affect B cell (1.03% vs. 0.87%) and Treg (0.76% vs. 0.46%) representations in the mice with HP, maintaining their levels above both the main control and control levels.

The 1,25(OH)_2_-VD3 treatment significantly reduced the excessive accumulation of neutrophils (1.01% vs. 34.47%), lymphocytes Tc (6.31% vs. 9.36%), lymphocytes Th (4.14% vs. 11.23%), and dendritic cells (3.75% vs. 9.20%) induced by the VD3-deficient mice’s exposure to the antigen of *P. agglomerans*. The 1,25(OH)_2_-VD3 intervention in the SE-PA + 1,25(OH)2-VD3 research group restored the physiological level of lymphocytes Tc, while in the case of neutrophils, lymphocytes Th, and dendritic cells, it inhibited their influx to pulmonary compartments below values observed in healthy animals on a VD3-sufficient diet. 1,25(OH)_2_-VD3 also inhibited the influx of macrophage M1 to the lungs of the mice with HP (0.58% vs. 10.09%), below the main control levels. On the contrary, the 1,25(OH)_2_-VD3 intervention additionally intensified the excessive inflow of Treg cells in the mice with HP (0.82% vs. 0.46%). At the same time, the mice with HP’s exposure to 1,25(OH)_2_-VD3 did not cause any changes in the amounts of B cells (0.75% vs. 0.87% in SE-PA) and M2 macrophages (1.73% vs. 2.79% in SE-PA).

### 2.4. Inhaled VD3 Metabolites Silenced EMT at the mRNA Level in VD3-Deficient Mice with HP

**Figure 4:**
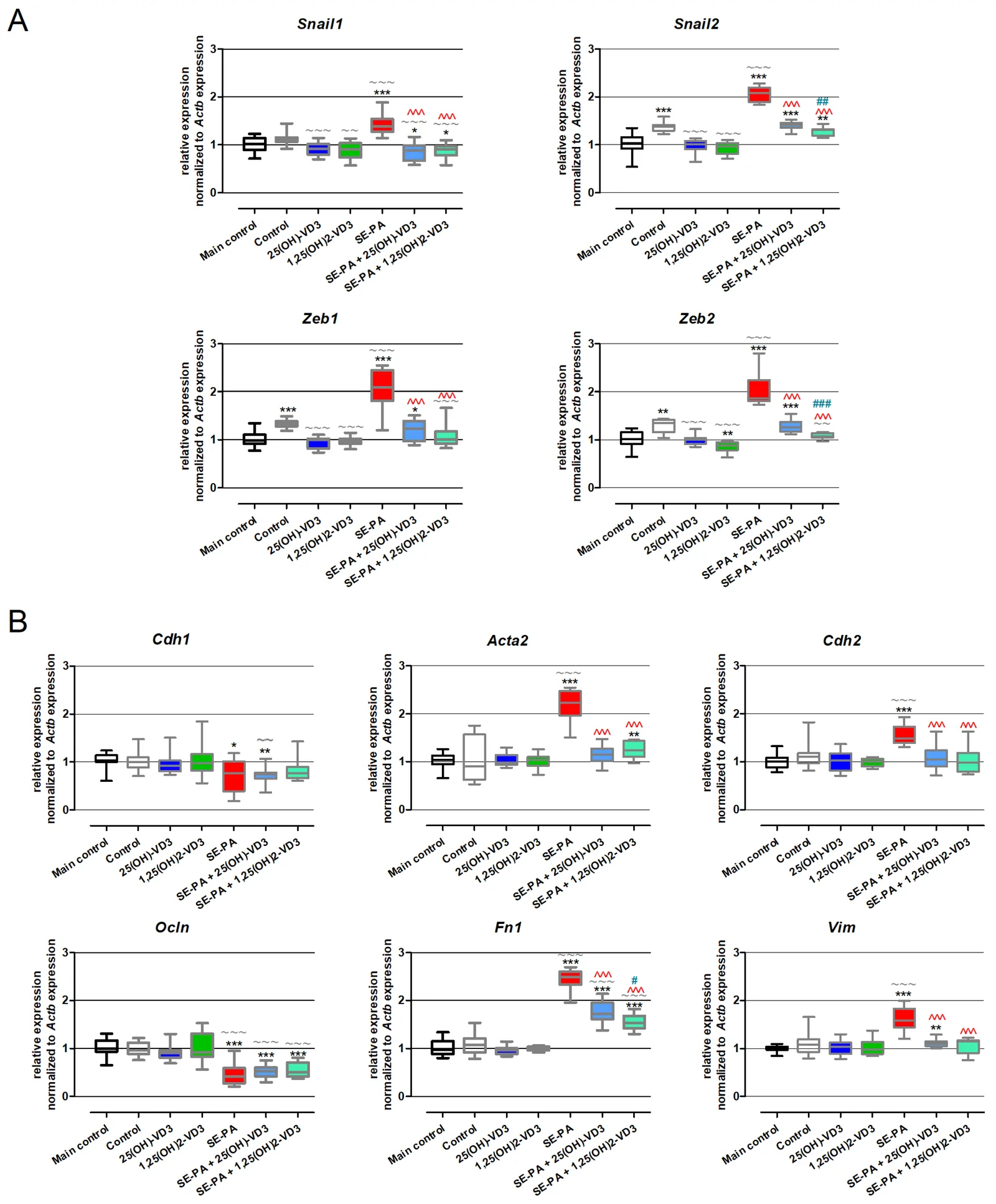
Alterations in the gene expression of the EMT markers observed in the VD3-deficient mice with HP using 25(OH)-VD3 or 1,25(OH)_2_-VD3. The gene expression in lung homogenates was examined by real-time PCR. (**A**) The relative gene expression of the EMT transcription factors. (**B**) The relative gene expression of the epithelial and mesenchymal cell markers. Statistical significance was examined using the Wilcoxon–Mann–Whitney test with Benjamini–Hochberg correction. Statistical comparisons are presented for: * (vs. the main control); ~ (vs. the control); ^ (SE-PA + 25(OH)-VD3 vs. SE-PA and SE-PA + 1,25(OH)2-VD3 vs. SE-PA); and # (SE-PA + 25(OH)-VD3 vs. SE-PA + 1,25(OH)2-VD3). The number of symbols corresponds with the significance level: one (*p* < 0.05), two (*p* < 0.01), or three (*p* < 0.001). The numerical data are listed in Supplementary Table S4.

The expression of genes related to EMT (epithelial to mesenchymal transitions) recorded by RealTime PCR in the VD3-deficeint mice with HP (Figure 4 and Table S4) revealed up-regulation of the key EMT transcription factors (*Snail1*: 1.39, *Snail2*: 2.08, *Zeb1*: 2.10, and *Zeb2*: 1.86) and markers of mesenchymal cells (*Acta2*: 2.23, *Cdh2*: 1.48, *Fn1*: 2.49, and *Vim*: 1.59), and the observed changes were statistically significant compared to both the main control and control animals. Animals on a VD3-deficient diet who inhaled the antigen of *P. agglomerans* also showed down-regulated expression of epithelial cell markers (*Cdh1*: 0.77 and *Ocln*: 0.42); the observed changes were significant compared to the main control, while in the case of *Ocln*, it was also comparable to the control mice.

In mice with HP, inhalation of 25(OH)-VD3 significantly reduced the over-expressed, by both disease development and VD3-deficient diet, amounts of both the EMT-transcription factors and mesenchymal cell markers. The 25(OH)-VD3 treatment restored the physiological levels of *Acta2* (1.15) and *Cdh2* (1.05). Nevertheless, the expression of *Snail2* (1.42 vs. 1.39 in the control), *Zeb1* (1.24 vs. 1.34 in the control), *Zeb2* (1.26 vs. 1.35 in the control), and *Vim* (1.11 vs. 1.08 in the control) recorded in the mice with HP that inhaled 25(OH)-VD3 to the levels observed in untreated mice on the same deficient diet. Among the investigated genes, the most sensitive to the 25(OH)-VD3 treatment was *Snail1* (0.89), the expression of which in SE-PA + 25(OH)-VD3 mice was significantly lower than that noted in both the main control and control animals, while the most resistant to the beneficial impact of the 25(OH)-VD3 treatment was *Fn1* (1.72), the expression of which, even after therapeutic intervention, was higher than that observed in healthy animals. On the contrary, the comparison data collected from the VD3-deficient mice with HP before and after exposure to 25(OH)-VD3 revealed that the expression of epithelial cell markers did not change in response to the treatment (*Cdh1*: 0.77 vs. 0.74 and *Ocln*: 0.42 vs. 0.53).

Similar to 25(OH)-VD3, the bioactive form of VD3 also inhibited gene expression of all of the examined EMT transcription factors and mesenchymal cell markers in the VD3-deficient mice with HP; however, the pattern of change was a little different. Treatment with 1,25(OH)_2_-VD3 restored the physiological levels of *Zeb1* (1.01), *Zeb2* (1.07), *Cdh2* (0.99) and *Vim* (1.15), or at least reduced the over-expression caused by disease development of *Snail2* (1.32 vs. 1.39 in the control) and *Acta2* (1.24 vs. 0.91 in the control) to the levels recorded in healthy VD3-deficient mice. The most significant silencing of up-regulated disease expression was observed in the case of *Snail1* (0.90), in which the SE-PA + 1,25(OH)_2_-VD3 group was well below both the main control and control groups. At the same time, the overexpression of *Fn1* (1.53) in mice with HP was significantly inhibited by the inhalation of 1,25(OH)_2_-VD3, but the amount was still higher than that recorded in healthy animals. The inhalation of 1,25(OH)_2_-VD3 did not impact the expression of epithelial cell markers in mice with HP; the expression of *Cdh1* (0.76) was maintained at both the main control and control levels.

### 2.5. Inhaled VD3 Metabolites Silenced EMT for Protein Level in VD3-Deficient Mice with HP

**Figure 5:**
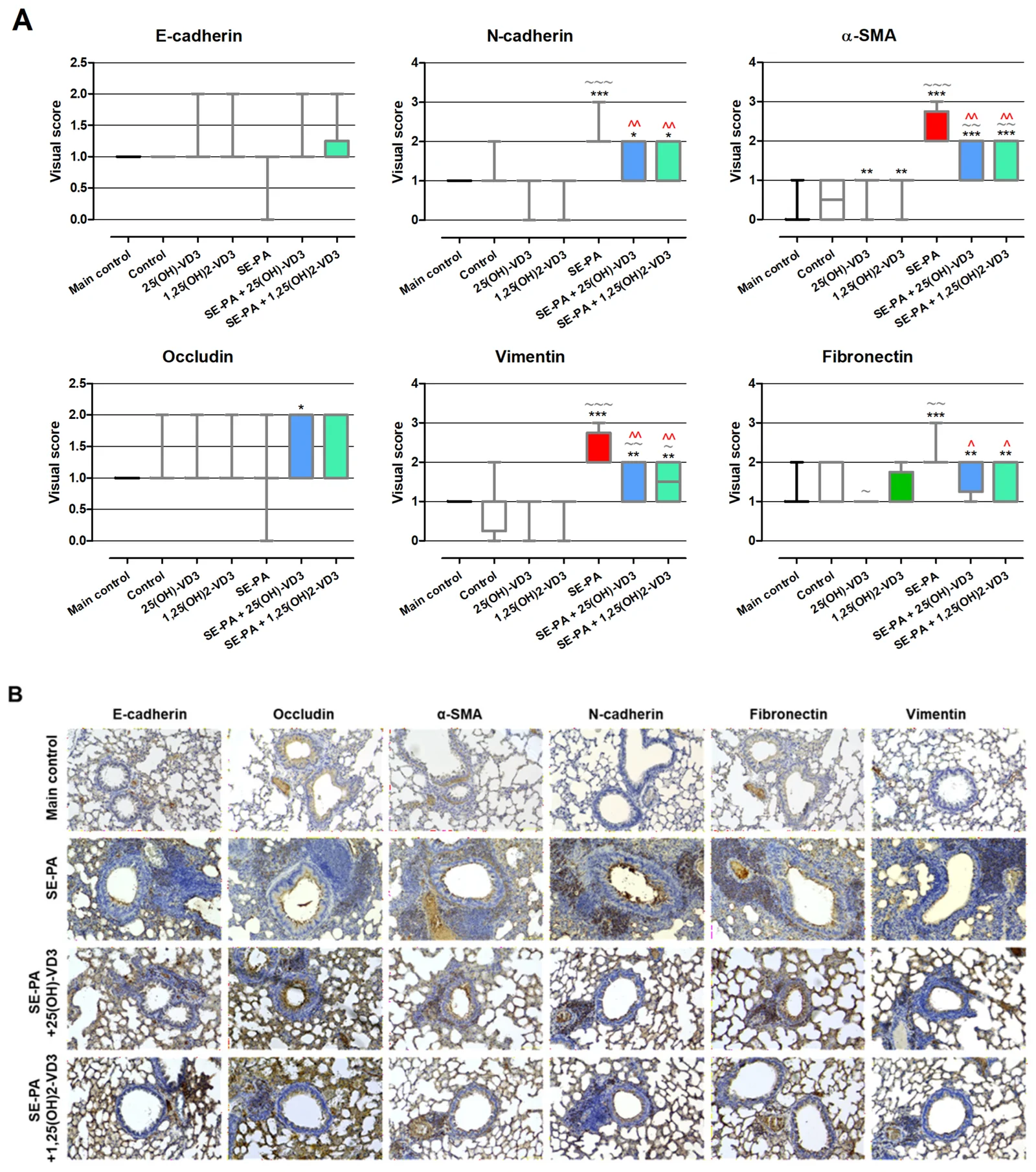
(**A**). Alterations in the expression of EMT markers observed in the VD3-deficient mice with HP treated with 25(OH)-VD3 or 1,25(OH)_2_-VD3. (**A**) The visual quantification of the protein expression on immunohistochemically stained murine lung tissue sections. The visual scores (five-stage system, 0–4), as acquired by a pathologist, are presented. Statistical significance was examined using the Wilcoxon–Mann–Whitney test with Benjamini–Hochberg correction. Statistical comparisons are presented for: * (vs. the main control); ~ (vs. the control); and ^ (SE-PA + 25(OH)-VD3 vs. SE-PA or SE-PA + 1,25(OH)2-VD3 vs. SE-PA). The number of symbols corresponds with the significance level: one (*p* < 0.05), two (*p* < 0.01), or three (*p* < 0.001). The numerical data are listed in Supplementary Table S5. (**B**) Representative photographs of lung sections stained with antibodies specific for EMT markers captured under a light microscope at 100× magnification.

As presented in Figure 5A and Table S5, histopathologist examination revealed that the VD3-deficient mice with HP have increased amounts of the mesenchymal cell markers N-cadherin, fibronectin, α-SMA, and vimentin (median of visual scores was 2 for all indicated proteins), contrary to the amount of epithelial cell markers maintained at levels observed in the untreated mice on both the VD3-sufficent and VD3-defficient diets (median of visual scores was 1 for all of the indicated proteins).

The treatment of mice with HP with 25(OH)-VD3 lowered the overexpression of mesenchymal cell markers; only the amounts of N-cadherin and fibronectin reached the levels recorded in the control group. On the contrary, the examined intervention did not impact the expression of epithelial cell markers: E-cadherin and occludin. The median of visual scores assigned by a pathologist for EMT markers after 14 days in the mice with HP that inhaled 25(OH)-VD3 were as follows: E-cadherin (1), occludin (1), α-SMA (1), N-cadherin (1), fibronectin (2), and vimentin (2).

The mice with HP that inhaled 1,25(OH)_2_-VD3 revealed a similar pattern of change to those recorded after the 25(OH)-VD3 treatment. The 1,25(OH)_2_-VD3 intervention did not impact E-cadherin and occludin expression, but significantly reduced the levels of mesenchymal cell markers. Similar to its precursor, the 1,25(OH)_2_-VD3 treatment restored the control amounts of N-cadherin and fibronectin. The median of the visual scores assigned by a pathologist for EMT markers after 14 days of inhalation of 1,25(OH)_2_-VD3 by mice with HP were as follows: E-cadherin (1), occludin (1), α-SMA (1), N-cadherin (1), fibronectin (2), and vimentin (1.5).

### 2.6. Inhaled VD3 Metabolites Modulated the Expression of the Factors Relevant to Tissue Repair During the Development of HP in VD3-Deficient Mice

**Figure 6:**
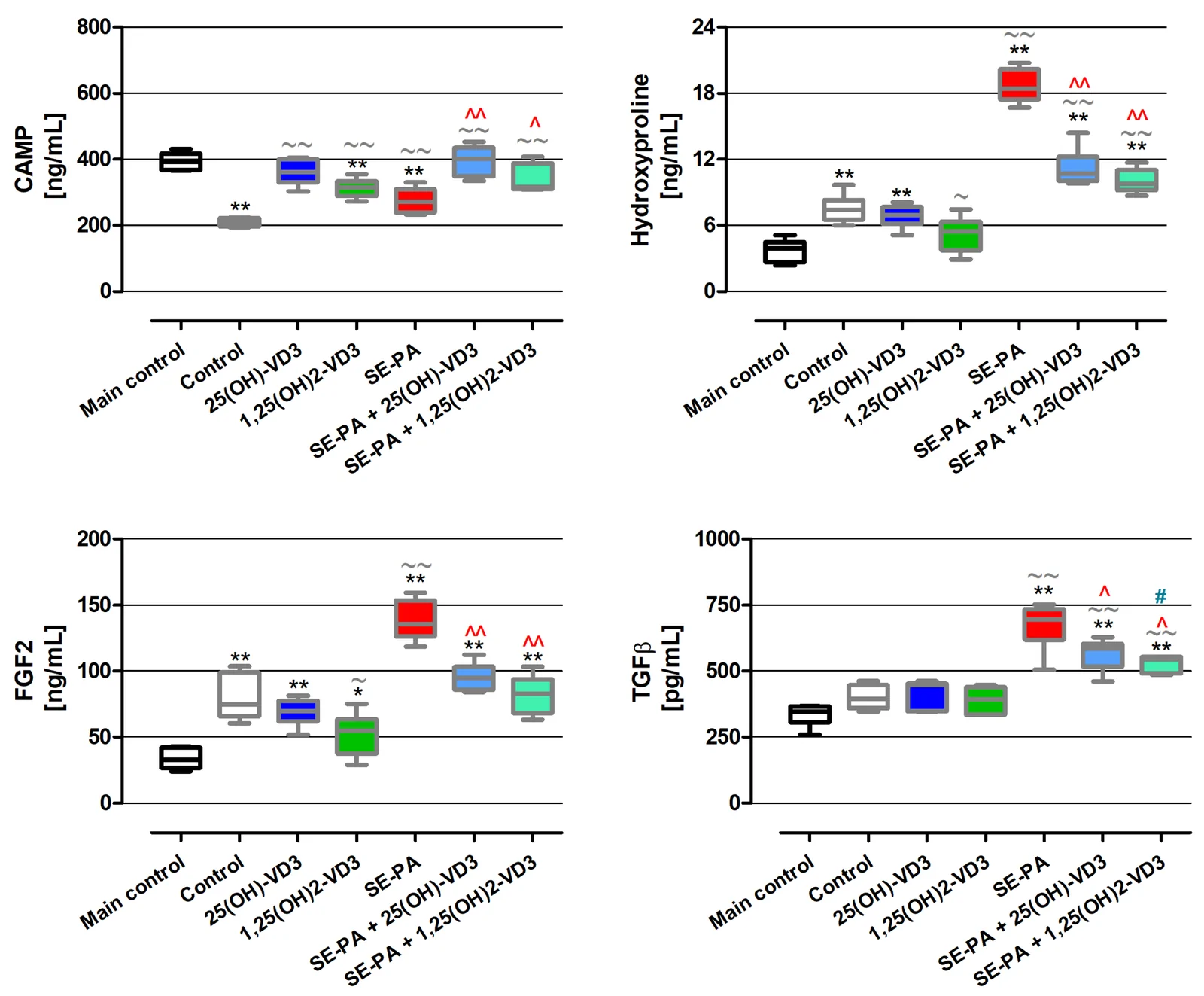
Alterations in the expression of fibrosis markers and factors regulating tissue repair in the VD3-deficient mice with HP in response to 25(OH)-VD3 or 1,25(OH)_2_-VD3. The protein concentrations were determined in lung homogenates by using dedicated ELISA kits. Statistical significance was examined using the Wilcoxon–Mann–Whitney test with Benjamini–Hochberg correction. Statistical comparisons are presented for: * (vs. the main control); ~ (vs. the control); ^ (SE-PA + 25(OH)-VD3 vs. SE-PA and SE-PA + 1,25(OH)2-VD3 vs. SE-PA); and # (SE-PA + 25(OH)-VD3 vs. SE-PA + 1,25(OH)2-VD3). The number of symbols corresponds with the significance level: one (*p* < 0.05), two (*p* < 0.01). The numerical data are listed in Supplementary Table S6.

As presented in Figure 6 and Table S6, HP development in the VD3-defficient mice significantly increased the fibrosis marker hydroxyproline (18.43 ng/mL vs. 3.92 ng/mL in the main control), the factors promoting pathological tissue repair FGF2 (135.74 ng/mL vs. 32.64 ng/mL in the main control), and TGFβ (695.23 pg/mL vs. 346.53 pg/mL in the main control), and simultaneously inhibited the amount of pro-healing antimicrobial peptides CAMP (271.28 ng/mL vs. 391.51 ng/mL in the main control). The discovered changes were significant compared to both the untreated mice on the VD3-sufficient and VD3-deficient diets.

The treatment of mice with HP with 25(OH)-VD3 inhibited the negative changes caused by antigen exposure. 25(OH)-VD3 administered together with SE-PA elevated the amount of CAMP (400.95 ng/mL vs. 271.28 ng/mL) to its physiological level (391.51 ng/mL). At the same time, the 25(OH)-VD3 treatment decreased the overexpression of hydroxyproline (10.69 ng/mL vs. 18.43 ng/mL), FGF2 (95.00 vs. 135.74 ng/mL), and TGFβ (584.90 pg/mL vs. 695.23 pg/mL), but the tested intervention was not able to restore the physiological levels of the indicated factors.

The inhalation of 1,25(OH)_2_-VD3 by mice with HP revealed a similar pattern of favourable changes to those previously described in the case of 25(OH)-VD3. The bioactive form of VD3 restored physiological levels of CAMP in the VD3-deficient mice with HP (316.82 ng/mL vs. 391.51 ng/mL). Moreover, 1,25(OH)_2_-VD3 administered together with SE-PA significantly reduced the elevated amounts of hydroxyproline (9.76 ng/mL vs. 18.43 ng/mL), FGF2 (82.80 vs. 135.74 ng/mL), and TGFβ (543.85 pg/mL vs. 695.23 pg/mL). Despite the beneficial impact of the 1,25(OH)_2_-VD3 treatment on mice with HP, the tested compound was not able to completely neutralise the negative influence of the VD3-deficient diet and *P. agglomerans* exposure.

Comparison of the concentration of proteins involved in regeneration with fibrosis scores across all of the research groups (Figure S1) revealed positive correlations with the pathological repair process and fibrosis inducers (hydroxyproline, TGFβ, and FGF2). At the same time, there was a lack of correlation between fibrosis scores and CAMP. The relationships between factors regulating the wound healing process and fibrosis score were characterised by Spearman’s rho: hydroxyproline 0.70 (*p* < 0.001), FGF2 0.69 (*p* < 0.001), and TGFB 0.56 (*p* < 0.001).

### 2.7. Inhaled VD3 Metabolites Restored Serum but Not Pulmonary Levels of Calcitriol in VD3-Deficient Mice with HP

**Figure 7:**
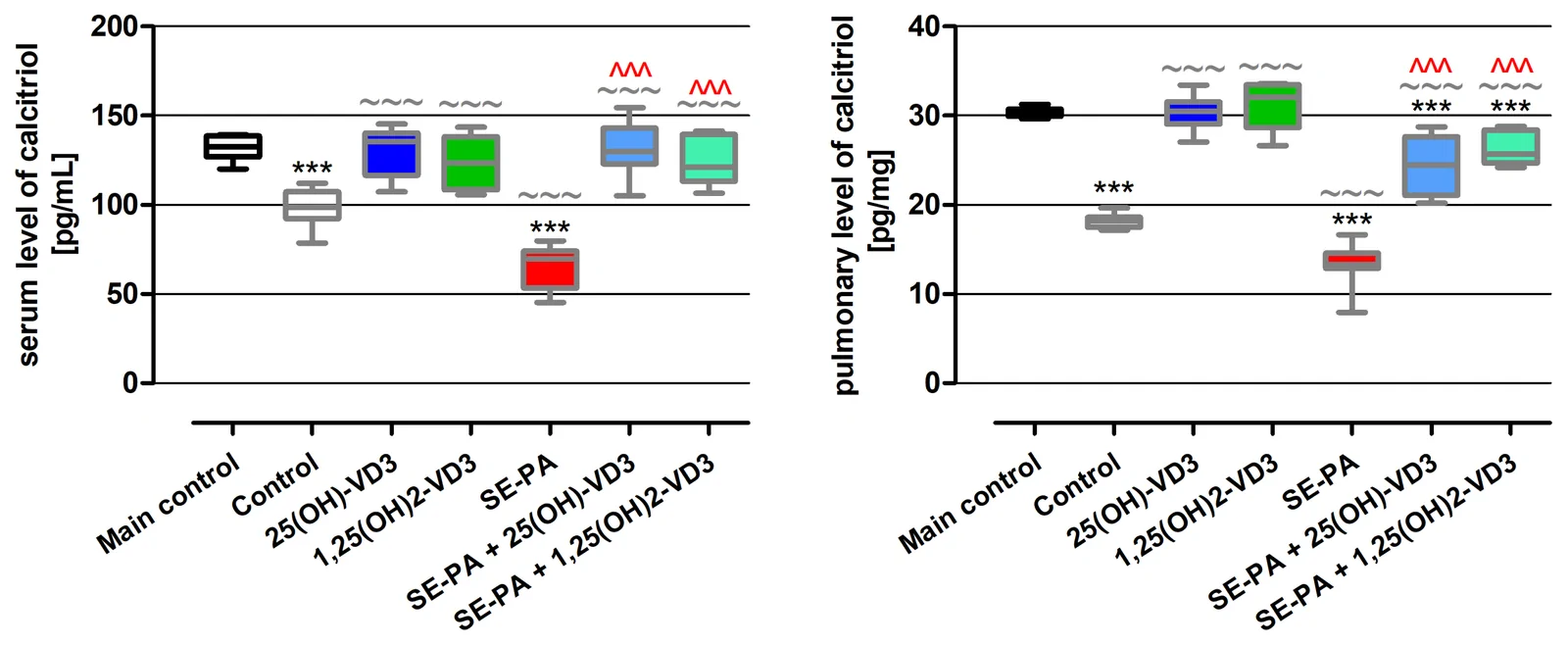
Changes in the serum and pulmonary levels of calcitriol in response to the VD3-deficient mice with HP to treatment with 25(OH)-VD3 or 1,25(OH)_2_-VD3. The calcitriol concentrations in serum and lung tissue homogenates were determined by dedicated ELISA. Statistical significance was examined using the Wilcoxon–Mann–Whitney test with Benjamini–Hochberg correction. Statistical comparisons are presented for: * (vs. the main control); ~ (vs. the control); and ^ (SE-PA + 25(OH)-VD3 vs. SE-PA and SE-PA + 1,25(OH)2-VD3 vs. SE-PA). The number of symbols corresponds with the significance level: three (*p* < 0.001). The numerical data are listed in Supplementary Table S7.

As presented in Figure 7 and Table S7, HP development additionally lowered endogenous serum and pulmonary concentrations of calcitriol, decreased by a cholecalciferol-restricted diet. Serum levels of calcitriol in the SE-PA, control, and main control research groups were as follows: 69.86 pg/mL, 98.64 pg/mL, and 132.71 pg/mL. Pulmonary levels of calcitriol in the above-mentioned research groups were as follows: 13.31 pg/mg, 18.21 pg/mg, and 30.21 pg/mg.

The 25(OH)-VD3 intervention significantly increased both the serum and pulmonary levels of calcitriol. Nevertheless, the inhalation of 25(OH)-VD3 by the mice with HP restored the physiological level of calcitriol only in the serum (130.08 pg/mL vs. 132.71 pg/mL in the main control). The pulmonary level of calcitriol improved in the mice with HP treated with 25(OH)-VD3, rising above the control level but not reaching the physiological value (24.50 pg/mg vs. 18.21 pg/mg vs. 30.21 pg/mg).

The inhalation of 1,25(OH)_2_-VD3 by the mice with HP also beneficially impacted the serum level of calcitriol, raising its value to a physiological concentration (121.19 pg/mL vs. 132.71 in the main control). Despite a significant increase in the pulmonary amount of calcitriol in the mice with HP exposed to this metabolite, its level after the intervention was higher than that recorded in the control animals, but still lower than that noted in the main control mice (25.71 pg/mg vs. 18.21 pg/mg vs. 30.21 pg/mg).

Comparison of the calcitriol concentration with fibrosis scores across all of the research groups (Figure S2) revealed negative correlations. The relationship between the serum level of calcitriol and fibrosis score was characterised by Spearman’s rho, −0.24 (*p* = 0.045). Similarly, the relationship between the pulmonary level of calcitriol and the fibrosis score was characterised by Spearman’s rho, −0.64 (*p* < 0.001).

### 2.8. Principal Component Analysis Reveals Coordinated Multivariate Response to VD3 Metabolite Treatment in VD3-Deficient Mice with HP

**Figure 8:**
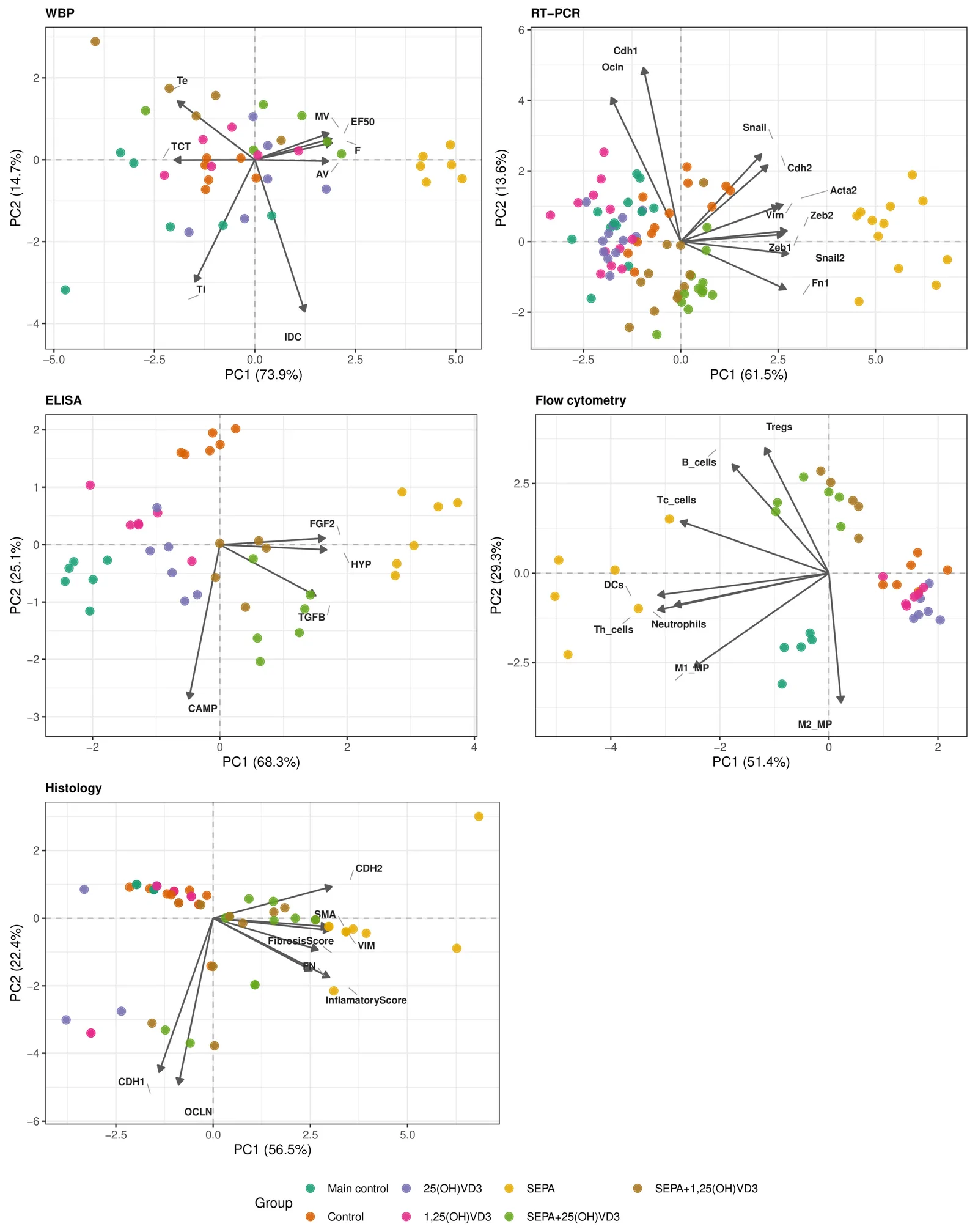
Principal component analysis biplots for the five analytical layers. Principal component analysis was performed separately for whole-body plethysmography, RT-PCR gene expression, ELISA-based tissue repair factors, flow cytometry, and immunohistochemical assessment of EMT markers. Each biplot displays sample scores for PC1 and PC2 (coloured by research group) together with variable loading vectors. The percentage of the total variance explained by each component is indicated on the respective axis. The variables were centred and scaled to unit variance prior to the analysis. Arrow lengths represent the squared cosine values (cos^2^) for each variable, with higher values indicating a more substantial association with the respective principal components.

To assess whether the individual changes described above reflect a coordinated biological response, PCA was performed separately for each analytical layer. Biplots are presented in Figure 8. In all five analytical layers, PC1 and PC2 jointly explained 75–93% of total variance (ELISA: 93.4%; WBP: 88.6%; flow cytometry: 80.7%; histology: 78.9%; and RT-PCR: 75.1%), confirming tight co-regulation of variables within each domain. PC1 consistently captured the disease–treatment gradient. In WBP, PC1 (73.9%) separated the restrictive breathing pattern (F, EF50, MV, and AV) from normal respiratory cycle parameters (TCT, Ti, and Te). In RT-PCR, PC1 (61.5%) represented the EMT axis—mesenchymal factors (*Acta2*, *Cdh2*, *Fn1*, *Snail1*, *Snail2*, *Vim*, *Zeb1*, and *Zeb2*) loaded in one direction, and epithelial markers (*Cdh1* and *Ocln*) in the opposite direction. Notably, *Cdh1* and *Ocln* dominated PC2 (13.6%), forming a dimension independent of the mesenchymal axis, consistent with the inability of the treatment to restore epithelial markers (Section 2.4). In ELISA, PC1 (68.3%) captured the co-regulated fibrosis signature (hydroxyproline, FGF2, and TGFβ), while CAMP loaded almost exclusively on PC2 (25.1%), indicating that CAMP restoration is largely independent of profibrotic mediator suppression. In flow cytometry, PC1 (51.4%) reflected lymphocyte infiltration (neutrophils, Th, Tc, B cells, Tregs, and DCs), while PC2 (29.3%) separated macrophage polarisation (M1 and M2). In histopathological assessment, PC1 (56.5%) integrated tissue damage and mesenchymal markers, while PC2 (22.4%) again captured the epithelial content (OCLN—occludin and CDH1—E-cadherin), mirroring the RT-PCR pattern. Across all of the analytical layers, the SE-PA mice occupied the disease pole of PC1, the healthy controls clustered at the opposite end, and the VD3-treated groups occupied intermediate positions, with the 1,25(OH)_2_-VD3-treated group consistently closer to the healthy controls than the 25(OH)-VD3-treated group.

### 2.9. Direct Comparison Confirms Significantly Stronger Immunomodulatory and EMT-Suppressive Effects of 1,25(OH)_2_-VD3 Relative to 25(OH)-VD3

To formally test whether the numerical differences between 25(OH)-VD3 and 1,25(OH)_2_-VD3 described above translate into a significant advantage of one metabolite over the other, a direct pairwise comparison (SE-PA + 1,25(OH)2-VD3 vs. SE-PA + 25(OH)-VD3) was performed for all 40 parameters presented in Figure 1, Figure 2, Figure 3, Figure 4, Figure 5, Figure 6 and Figure 7 (the Wilcoxon–Mann–Whitney test with Benjamini–Hochberg correction, consistent with the statistical framework used throughout this study; the full results are in Supplementary Table S8). This comparison confirmed that 1,25(OH)_2_-VD3 produced a significantly stronger suppression of pulmonary neutrophil (1.01% vs. 2.29%; *p* = 0.008) and Tc lymphocyte (6.31% vs. 8.02%; *p* = 0.042) infiltration, as well as the expression of the EMT-related genes *Zeb2* (1.07 vs. 1.26; *p* < 0.001) and *Snail2* (1.32 vs. 1.42; *p* = 0.01) compared with 25(OH)-VD3. Indicated comparisons have shown *Fn1* and TGFβ were significant at raw *p* (*p* < 0.05), but not after BH correction (*Fn1*: 1.53 vs. 1.72; *p* = 0.052, TGFβ: 543.85 pg/mL vs. 584.90 pg/mL; *p* = 0.053); thus, they are shown as non-significant. For the remaining 36 parameters, including both the inflammation and fibrosis scores, all of the respiratory parameters, and the hydroxyproline concentration, as well as the expression of epithelial and mesenchymal cell markers, the numerical advantage of 1,25(OH)_2_-VD3 did not reach statistical significance at the current sample size. Notably, for CAMP, the Inspiratory Duty Cycle, and *Acta2*, the numerical direction favoured 25(OH)-VD3, although these differences were likewise not statistically significant. These findings indicate that the superior therapeutic potential of 1,25(OH)_2_-VD3 is most robustly confirmed at the level of immune cell infiltration and EMT-transcriptional regulation, while its advantage for downstream structural and functional outcomes remains a consistent trend rather than a statistically established difference.

## 3. Discussion

The aim of the presented studies was the examination of the effectiveness of therapeutic interventions based on the inhalation of 25(OH)-VD3 and 1,25(OH)_2_-VD3 during the acute phase of hypersensitivity pneumonitis in VD3-deficient mice. To better understand all aspects of the presented studies, including the decision to focus on VD3-deficient mice, as well as choosing the direct administration of VD3 metabolites to the respiratory tract rather than their oral administration, the most important findings of our earlier research are presented below.

First, our earlier studies clearly demonstrated that dietary cholecalciferol restriction, causing VD3 deficiency, significantly impacts lung anatomy and physiology, promoting the development of pulmonary fibrosis in response to chronic exposure to the *P. agglomerans* antigen. Increased susceptibility to the development of pulmonary fibrosis was associated with the intensification of EMT, a key mechanism underlying fibrosis, as evidenced by the overexpression of EMT transcription factors (*Snail2*, *Zeb1*, and *Zeb2*) and mesenchymal markers (*Cdh2*/N-cadherin, *Fn1*/fibronectin, *Vim*/vimentin, and *Acta2*/α-SMA). The impaired tissue repair observed in the mice with HP with VD3 deficiency correlated with increased infiltration of immune cells into the lung parenchyma (mainly neutrophils, macrophages, dendritic cells, and lymphocytes Tc), increased production of profibrotic growth factors (FGF2 and TGFβ), and a significant deterioration in respiratory parameters (F—frequency of breathing, TCT—Total Cycle Time, Ti—time of inspiratory, Te—time of expiratory, MV—minute volume, and EF50—mid-tidal expiratory flow). Furthermore, the performed studies revealed that the exacerbated development of pulmonary fibrosis due to a cholecalciferol-restricted diet correlated with a deficit of endogenous pulmonary amount of calcitriol [17]. The discovery of a connection between dietary VD3-deficiency and the worsening of HP (particularly fibrotic manifestations) suggested the possibility of preventing pulmonary fibrosis through VD3 supplementation. However, the observed decrease in calcitriol levels in the lungs of the mice with HP on a standard VD3-sufficient diet indicated the need to base dietary recommendations on the level of this metabolite in the respiratory tract [17]. Ignoring this aspect may explain the considerable discrepancies in the effectiveness of VD3-based therapies for fibrotic lung diseases [33,34,35,36,37]. At the same time, the main reasons for the failure of these therapies included comorbidities and/or metabolic disorders affecting calcitriol synthesis, leading to its deficiency [33,35]. Taking this into account, as well as the risk of VD3 overdose leading to hypercalcaemia, hypercalciuria, or hyperphosphataemia, and, in the case of their non-treatment, to kidney stones, vascular calcification, or organ parenchyma calcification [36], we decided to base our strategy for preventing pulmonary fibrosis on VD3 metabolites delivered directly to the respiratory tract in doses, restoring physiological calcitriol levels in the lungs of animals with dietary VD3-deficiency [18,19,38]. The research was conducted using both 25(OH)-VD3 and 1,25(OH)_2_-VD3. The data were collected at two time points—after 14 and 28 days of daily inhalation of the *P. agglomerans* antigen and/or VD3 metabolites. The indicated research design allowed for tracking the treatment effects during both the acute (day 14 of the experiment) and chronic (day 28 of the experiment) phases of HP. The performed studies revealed that the inhalation of25(OH)-VD3 and 1,25(OH)_2_-VD3 effectively alleviated pathological changes induced by both dietary VD3-deficiency and antigen exposure, when harmful factors and therapeutic compounds are provided simultaneously [18,19]. The increased percentage of neutrophils, dendritic cells, and B cells induced by a deficient diet and 14 days of *P. agglomerans* antigen exposure was significantly reduced in response to both investigated metabolites. The beneficial effects of inhaled 25(OH)-VD3 and 1,25(OH)_2_-VD3 were associated with inhibition of EMT, as indicated by the reduced expression of EMT-related factors (*Snail1*, *Snail2*, *Zeb1*, *Zeb2*, *Nfkb*, and *TGFβ*) and mesenchymal markers (*Acta2*, *Cdh2*, *Fn1*,and *Vim*), as well as restoration of suppressed epithelial markers (*Cdh1* and *Ocln*). At the proteome level, the inhibition of the pathological deposition of extracellular matrix components (collagen, fibronectin, hydroxyproline, and vimentin) was observed, along with reduced overproduction of FGF2 and TGFβ. Inhalation of VD3 metabolites also stabilised respiratory parameters (F, TV, and MV) disrupted by HP progression. Histological analyses confirmed the beneficial effects of the tested metabolites on lung structure, evidenced by a significant reduction in inflammatory and fibrosis responses [18,19]. On day 28 of the experiment, the beneficial effects of 25(OH)-VD3 and 1,25(OH)_2_-VD3 inhalations on pulmonary fibrosis development in the VD3-deficient mice were associated with reduced infiltration of macrophages, neutrophils, lymphocytes Th, and lymphocytes Tc (both metabolites), as well as dendritic cells and B cells (1,25(OH)_2_-VD3). Histopathological analysis confirmed the suppression of inflammation and decreased collagen deposition responsible for the thickening of the alveolar septa. The antifibrotic effects of inhaled VD3 metabolites were associated with EMT inhibition. At the mRNA level, this was reflected by the down-regulation of transcription factors (*Snail1*, *Snail2*, *Zeb1*, *Zeb2*, *Nfkb*, and *Tgfβ*) and mesenchymal cell markers (*Acta2*, *Cdh2*, *Fn1*, and *Vim*), along with the up-regulation of epithelial cell markers (*Cdh1* and *Ocln*). At the proteome level, the beneficial effects of the tested metabolites were associated with the reduced over-production of TGFβ and FGF2, as well as the decreased deposition of fibronectin, vimentin, and hydroxyproline. As a consequence of the therapeutic effects of the investigated metabolites, respiratory parameters (F, MV, EF50, Ti, and Te) disrupted by both HP and VD3-deficiency were stabilised [17,18].

What is important is that the above-mentioned studies proved a correlation between pulmonary levels of calcitriol and the degree of fibrosis, confirming the main hypotheses of our research, that maintaining a physiological level of calcitriol is crucial for preventing pathological tissue repair observed in HP [17,18]. Despite the discovery of the therapeutic potential of inhaled VD3 metabolites, the important limitation of the described research was the moment of the initiation of therapy, which coincides with the beginning of HP–antigen exposure and does not reflect reality. Clinical observations clearly show that patients with HP present to a doctor in two main stages of HP development—at the acute or chronic phases. Fortunately, the overwhelming majority of patients with HP seek medical attention during the acute phase with flu-like symptoms before the appearance of the first signs of fibrosis, which allows for effective therapy preventing pathological tissue repair. Patients at the chronic phase with developed fibrosis can only count on alleviating the symptoms of the disease and stopping its further progression—fibrosis is an irreversible process [3,5,6,7,39]. Because of the indicated specificity of HP management, it seems reasonable to verify the therapeutic potential of inhaled 25(OH)-VD3 and 1,25(OH)_2_-VD3, beginning the intervention at the acute stage of the disease. To achieve this goal, inhalation of VD3 metabolites started after 14 days of daily exposure of mice to the antigen of *P. agglomerans* and was continued for the next 14 days, during which mice’s exposure to the fibrosis-inducer was continued. This strategy better reproduced the potential treatment regimen in laboratory conditions.

Another important issue which should be clarified before discussion of the presented results is the fact that this study was performed on VD3-deficient mice. There were two main reasons why the possibility of using inhalation with VD3-metabolites for the prevention of HP fibrosis was examined under VD3 deficiency instead of VD3-sufficient conditions. As revealed in our earlier studies, there is a correlation between pulmonary levels of calcitriol and the range of fibrotic changes over the course of HP [17,18,19]. The greater susceptibility of the VD3-deficient mice than the VD3-sufficient mice to fibrosis development with HP, discovered by our research team, may explain the immunoenhancement features of 1,25(OH)_2_-VD3, the deprivation of which promotes a more pronounced inflammatory response disturbing the proper repair of tissues and its orientation towards fibrosis [40,41]. The indicated discoveries suggest that restoring physiological levels of calcitriol in the respiratory tract by delivering it or its precursor directly to the airway may be the key to preventing pulmonary fibrosis. Furthermore, changes in lung function and the immune system observed in healthy mice on a cholecalciferol-restricted diet suggest that achieving a positive response to therapy will be more challenging herein than in mice on a standard diet, further supporting the validity of conducting the analyses under these conditions. The second reason why we decided to focus on VD3-deficient animals is the global pandemic of VD3 deficiencies with serious health consequences, including chronic respiratory diseases with fibrosis [33,35,36,42,43]. The prevalence of VD3 deficiency worldwide suggests the need to conduct research under such conditions, since they are typical for a significant part of the population [36,43].

Last but not least, the issue that requires clarification is the selection of animal gender. Due to the pioneering nature of this research, we decided to initially conduct it exclusively on males. The available data on the gender effect on VD3 metabolism and activity revealed that males have shown lower levels of 7-dehydrocholesterol and VD3 transporting protein (DBP), as well as higher expression of the enzyme CYP24A1 responsible for the catabolism of both 25(OH)-VD3 and 1,25(OH)_2_-VD3. On the contrary, females have shown higher levels of VDR and the enzyme CYP27B1, responsible for the 25(OH)-VD3 conversion to 1,25(OH)_2_-VD3. The indicated changes are associated with sex hormones, among which testosterone inhibits vitamin D metabolism, while oestrogen stimulates this process [44]. The cited data suggested that males may find it more difficult to achieve positive results in response to cholecalciferol supplementation. Consequently, selecting males to verify the effectiveness of therapy based on vitamin D3 supplementation seems to be a reasonable move. The selection of male mice was also supported by scientific reports, which revealed a more severe course of pulmonary fibrosis and a worse prognosis in men, especially older ones, than in women, which may be related to longer and/or more intense occupational exposure to antigens causing HP [45,46].

Taking all the above into account, the effect of the inhalation of VD3 metabolites (25(OH)-VD3 or 1,25(OH)_2_-VD3) on the course of pulmonary fibrosis in VD3-deficient mice during the acute phase of HP was examined. In the first step of the presented studies, the impact of VD3 metabolites on lung tissue morphology and function was assessed. Inhalation of VD3 metabolites significantly ameliorated histological manifestations of HP. Although both metabolites reduced inflammatory and fibrosis scores, 1,25(OH)_2_-VD3 reduced fibrosis to levels comparable with those of healthy animals, while 25(OH)-VD3 achieved a smaller, although still substantial, reduction. This numerical difference did not reach statistical significance in a direct pairwise comparison between the two treatment groups. These findings suggest that direct activation of the vitamin D receptor (VDR) may more effectively counteract pathological mechanisms driving pulmonary remodelling than inhalations with the precursor metabolite requiring local bioactivation [47]. Histopathological improvements were paralleled by the substantial restoration of respiratory function. Whole-body plethysmography revealed that HP development in the VD3-deficient mice induced a characteristic pattern of respiratory dysfunction manifested by increased frequency of breathing (F), minute volume (MV), accumulated volume (AV), mid-tidal expiratory flow (EF50), and Inspiratory Duty Cycle (IDC), accompanied by shortened respiratory cycle duration (TCT—Total Cycle Time) and both times of inspiration and expiration (Ti and Te). Such alterations were consistent with the progressive restriction of lung function resulting from enhanced immune cell infiltration and extracellular matrix accumulation, observed during histological evaluation, and correspond to respiratory abnormalities reported in experimental models of pulmonary fibrosis and restrictive lung disease [48,49]. Despite the fact that both examined metabolites aimed to alleviate respiratory disorders caused by HP, 25(OH)-VD3 restored the physiological level of three out of eight (EF50, IDC, and Te), and 1,25(OH)_2_-VD3 restored six out of eight (F, MV, AV, EF50, TCT, and Te) disturbed respiratory parameters. It should also be emphasised that inhalation of 1,25(OH)2-VD3 significantly lowered the IDC value, elevated by SE-PA, but in contrast to earlier presented data, the investigated therapy decreased the tested parameter below the main control level but lowered it further. Considering the biological significance of the elevated IDC level, which indicates the restriction of airflow, the effect of 1,25(OH)_2_-VD3 therapy should be interpreted cautiously as representing either complete normalisation, or even slight optimisation, of respiratory timing following attenuation of pulmonary fibrosis, rather than an unintended treatment-related effect. The discovered changes may reflect a robust local therapeutic response to inhaled 1,25(OH)_2_-VD3 combined with the normal biological variability associated with the relatively small sample size. In light of these data, we can conclude that mice with HP that inhaled 1,25(OH)_2_-VD3 improved in seven out of eight investigated respiratory parameters. 1,25(OH)_2_-VD3 restored a greater number of respiratory parameters to physiological levels than 25(OH)-VD3 (six vs. three of eight parameters relative to the main control); however, a direct pairwise comparison between the two treatment groups did not reach statistical significance for any individual WBP parameter. This trend is nonetheless consistent with the stronger antifibrotic properties attributed to the biologically active form of VD3 and VDR-mediated regulation of inflammatory and profibrotic pathways [47,50]. Furthermore, the results of the WBP and histological assessment suggested that suppression of pulmonary remodelling translated directly into a disorder of lung function, consistent with previous studies demonstrating a close relationship between the extent of tissue fibrosis and respiratory impairment in murine models of obstructive and restrictive respiratory diseases [49,51].

To better understand the discovered inhibition of immune cell infiltration into the lung tissue of the VD3-deficient mice with HP in response to inhalation of 25(OH)-VD3 and 1,25(OH)_2_-VD3, characteristic cell subpopulations were analysed using flow cytometry. The performed studies revealed profound alterations in pulmonary immune-cell composition during disease progression. The increased numbers of neutrophils and dendritic cells, as well as lymphocytes Th, Tc, Treg, and B cells, confirmed the establishment of a strong adaptive and innate immune response to the antigen of *P. agglomerans*. These findings are consistent with previous studies indicating that chronic exposure to microbial antigens induces extensive leukocyte recruitment and persistent alveolitis, which represents a central pathogenic mechanism of HP [5,6]. The accumulation of neutrophils and activated lymphocytes T contributes to ongoing tissue injury through the production of inflammatory mediators, reactive oxygen species, proteolytic enzymes, and profibrotic cytokines, including TGFβ, which elevates the amount of the latter and was also elevated in this study [6,52]. Importantly, the inhalation of both of the investigated VD3 metabolites significantly reduced pulmonary recruitment of neutrophils, lymphocytes Th, and dendritic cells, whereas 1,25(OH)_2_-VD3 additionally normalised the accumulation of lymphocytes Tc. Similar immunomodulatory effects of VD3 have been reported in experimental models of asthma, COPD, and pulmonary fibrosis, including chronic HP, where activation of the VDR suppresses antigen presentation by dendritic cells, inhibits activation of lymphocytes T, and reduces the production of pro-inflammatory cytokines, such as TNFα, IL6, and IFNγ [19,40,53,54]. Furthermore, several studies have demonstrated that VD3 signalling promotes a tolerogenic phenotype of dendritic cells characterised by the decreased expression of MHC-II and co-stimulatory molecules, thereby limiting the excessive activation of lymphocytes T [53,55]. Therefore, the reduced influx of inflammatory leukocytes observed in the present study likely reflects both direct and indirect immunoregulatory actions of VD3 metabolites. Interestingly, 1,25(OH)_2_-VD3 intervention further increased the accumulation of lymphocytes Treg in the mice with HP, whereas the 25(OH)-VD3 treatment had no significant effect on this cell population. This observation is in agreement with numerous reports demonstrating that 1,25(OH)_2_-VD3 promotes the differentiation, expansion, and functional activity of Treg cells [56,57]. Similar findings have been reported recently by our research team as an important mechanism of 1,25(OH)_2_-VD3 antifibrotic features in the murine HP model [19]. Another noteworthy observation concerns macrophage populations. The elevated proportion of pro-inflammatory M1 macrophages, accompanied by the reduced representation of reparative M2 in VD3-deficient mice in response to the antigen of *P. agglomerans*, indicates a persistent inflammatory state that may impair physiological tissue regeneration and promote fibrosis. Similar disturbances in macrophage polarisation have been described in chronic HP and other fibrotic lung diseases, including idiopathic pulmonary fibrosis (IPF), where sustained macrophage M1 activation contributes to inflammatory injury, while dysregulated macrophage M2 responses drive the deposition of the pathological extracellular matrix [58,59,60]. Thus, a proper balance between macrophage subsets is crucial for maintaining tissue homeostasis. In light of this, the data collected from the VD3-metabolites intervention are promising. Both inhalations with 25(OH)-VD3 and 1,25(OH)_2_-VD3 significantly reduced the increased amount of macrophage M1 in the mice with HP, which is consistent with their anti-inflammatory properties. Comparable effects have been reported in experimental models of pulmonary fibrosis, where VD3 signalling inhibited NFκB activation, reduced production of pro-inflammatory cytokines, and shifted macrophage polarisation towards a less inflammatory phenotype [53,61]. In addition to restoring the amount of M1 macrophages to the control level in response to both tested metabolites, the 25(OH)-VD3 treatment additionally lowered the amount of M2 macrophages, while the 1,25(OH)_2_-VD3 intervention did not affect this population. Nevertheless, comparison of the indicated results with the data collected from the untreated VD3-deficient mice revealed that the investigated interventions also maintained the amount of M2 macrophages at the control level. At first glance, the reduction in M2 macrophages might appear unfavourable because these cells participate in tissue repair; however, accumulating evidence suggests that excessive or prolonged activation of M2 macrophages may also contribute to fibrogenesis through the increased secretion of TGFβ, PDGF, and other profibrotic mediators [58,62]. Since M1 macrophages initiate the immune response in HP, while M2 macrophages coordinate tissue repair in response to antigen-induced injury, preservation of macrophage numbers by using VD3 metabolites may contribute to more balanced tissue regeneration and partially explain their antifibrotic effects observed previously. This interpretation is further supported by studies indicating that VD3 regulates macrophage plasticity and prevents the establishment of a profibrotic microenvironment within the lung [53]. Overall, the presented findings correspond with an increasing amount of evidence indicating VD3 as a key immunoregulator in chronic lung diseases with fibrosis [40]. The thus observed reduction in inflammatory-cell infiltration, enhancement of Treg-cell recruitment, and normalisation of macrophage polarisation collectively suggest that inhaled 25(OH)-VD3 and 1,25(OH)_2_-VD3 attenuate HP progression through the restoration of immune balance. Moreover, a direct pairwise comparison confirmed that 1,25(OH)_2_-VD3 produced significantly lower pulmonary neutrophil (*p* = 0.008) and Tc lymphocyte (*p* = 0.042) counts than 25(OH)-VD3, indicating that direct delivery of the biologically active form of VD3 provides superior control of chronic pulmonary inflammation at the cellular level and, consequently, more effective prevention of fibrosis.

Next to the restoration of immune homeostasis, the therapeutic effect of the 25(OH)-VD3 and 1,25(OH)_2_-VD3 interventions during the acute phase of HP was associated with the inhibition of EMT, which corresponds with previously described mechanisms of preventive action of these metabolites in HP fibrosis [18]; nevertheless, some discreet changes were noted. Both the 25(OH)-VD3 and 1,25(OH)_2_-VD3 treatments significantly attenuated up-regulated expression of EMT transcription factors (*Snail1*, *Snail2*, *Zeb1*, and *Zeb2*), as well as mesenchymal cell markers (*Acta2*, *Cdh2*, *Fn*, and *Vim*). Nevertheless, 1,25(OH)_2_-VD3 produced a more comprehensive effect, restoring physiological expression of *Zeb1*, *Zeb2*, *Cdh2,* and *Vim*, while substantially reducing expression of *Snail1*, *Snail2,* and *Acta2*. Instead, 25(OH)-VD3 was able to restore physiological gene expression levels of *Acta2* and *Cdh2*. Particularly notable was the profound suppression of Snail1 expression by both of the inhaled VD3 metabolites, reducing transcript levels below those observed in healthy animals. Since *Snail1* is considered a master regulator, initiating EMT programmes through repression of epithelial genes and activation of mesenchymal transcriptional networks, its strong inhibition may represent a key mechanism underlying the antifibrotic activity of the examined metabolites. Interestingly, neither 25(OH)-VD3 nor 1,25(OH)_2_-VD3 increased the fibrosis-suppressed expression of epithelial cell markers (*Cdh1* and *Ocln*), which is inconsistent with the previously reported preventive effects of these metabolites during HP fibrosis development, where the restoration of the indicated gene expression was recorded [18]. Immunohistochemistry, conducted as the next step, clearly demonstrated that the inhalation of VD3 metabolites did not impact the expression of E-cadherin and occludin, while significantly inhibiting the overexpressed amount of mesenchymal cell markers (N-cadherin, α-SMA, fibronectin, and vimentin). This observation suggests that the examined therapeutic interventions primarily inhibited the progression of EMT and mesenchymal differentiation, rather than fully reversing already established epithelial injury. Confirmed based on proteome levels, the lack of VD3-metabolites influence on the expression of epithelial cell markers, distinguishing the therapeutic intervention from previously examined preventive use of inhaled 25(OH)-VD3 and 1,25(OH)_2_-VD3, which included the enhancement of the amounts of E-cadherin and occludin silenced by fibrosis development [18]. The indicated discrepancy may be explained by differences between preventive and therapeutic intervention paradigms. In the preventive model, VD3 metabolites were administered before extensive epithelial damage and the fibrotic process had developed, enabling the preservation of the epithelial phenotype. In contrast, the present therapeutic protocol was initiated after disease establishment, when epithelial injury and the pathological process of their repair, leading to fibrosis, had already been activated. Consequently, the ability of 25(OH)-VD3 and 1,25(OH)_2_-VD3 to restore epithelial differentiation markers may be limited despite their capacity to suppress EMT-promoting signalling pathways.

Unfortunately, apart from previous studies by our team, there are no other reports on the influence of VD3 metabolites on pulmonary fibrosis in HP. However, in the context of EMT, it is worth recalling the observations of other researchers from one of the most important animal models of idiopathic pulmonary fibrosis (IPF), in which pathological changes were induced in C57BL/6 mice by a single intratracheal administration of the fibrosis-inducing agent, bleomycin. Due to the nature of the intervention, which, similarly to our study, occurred after the induction of fibrosis, it is worth mentioning the study by Zhu et al., who analysed the effect of daily 14-day intraperitoneal injections with cholecalciferol (2 μg/kg), initiated 14 days after bleomycin administration (2.5 mg/kg). This intervention resulted in the inhibition of fibrosis by limiting the undesirable deposition of extracellular matrix components (hydroxyproline, type I collagen, and α-SMA). Importantly, histological analyses also confirmed the reduced infiltration of inflammatory cells, oedema, and structural destruction of lung tissue, which is consistent with our observations [63]. A similar therapeutic effect of cholecalciferol in a murine model of bleomycin-induced IPF was reported by Tzilas et al. In that study, therapy with cholecalciferol administered orally (1 μg/kg) for 10 consecutive days was started on the third day after bleomycin administration (2.5 IU/kg). The performed studies revealed that VD3 partially reversed the fibrotic changes in mice with IPF, a sign of which was noted in the histological evaluation: decreased collagen deposition in the interstitium and perivascular and parenchymal spaces. The beneficial effect of oral administration of cholecalciferol was associated with the down-regulation of overexpressed genes coding collagen (*col1a1* and *col3a1*) and α-SMA (*acta2*). Simultaneously, a significant reduction in both hydroxyproline and α-SMA in mice with IPF in response to cholecalciferol treatment was also recorded [64]. Finally, it is worth mentioning the results from Chang et al., who analysed the effect of the active VD3 analogue paricalcitol over the course of IPF. In this study, paricalcitol therapy (150 ng/kg) was administered intraperitoneally for 26 consecutive days, beginning on the second day after fibrosis induction by bleomycin (1.5 Units/kg). Treatment with paricalcitol alleviated lung fibrosis thanks to the suppression of the fibrotic inducer TGFβ. The advantageous effect of the tested compound was also associated with the inhibition of extracellular matrix components elevated by IPF development. The paricalcitol treatment substantially blocked the induction of α-SMA, fibronectin, and collagen type I, II and III at both mRNA and protein levels [65].

The molecular findings described above were further supported by the analysis of soluble mediators involved in tissue repair and fibrogenesis. Examination focused on TGFβ, recognised as the central inducer of EMT, fibroblast activation, and extracellular matrix deposition during pulmonary fibrosis [66,67]. Additionally, FGF2 was assessed due to its established role in promoting fibroblast proliferation, myofibroblast differentiation, and collagen accumulation within the injured lung [67,68]. Moreover, hydroxyproline, considered the most widely used biochemical marker of collagen deposition and tissue fibrosis, was also evaluated [66]. As expected, HP progression significantly increased pulmonary concentrations of hydroxyproline, TGFβ, and FGF2, confirming ongoing collagen synthesis and the activation of pathological tissue repair. Similar overproduction of these profibrotic mediators has been reported in numerous experimental models of pulmonary fibrosis, where increased TGFβ signalling closely correlates with disease severity and progression [21,66,67]. Importantly, both 25(OH)-VD3 and 1,25(OH)_2_-VD3 significantly reduced the disease-induced elevation of these mediators, although complete normalisation of their concentration was not achieved. This partial restoration closely parallels the improvements observed in histological fibrosis scores and EMT-related gene and protein expression. These findings indicate that the examined therapeutic intervention attenuates, but does not eliminate, profibrotic processes. Comparable observations have been reported in previously described research by Chang et al., where suppression rather than complete elimination of TGFβ activity was sufficient to substantially attenuate fibrosis [65]. In addition to evaluating fibrosis-promoting factors, CAMP was assessed due to its documented antifibrotic properties [10,69] and its recognised role in coordinating tissue repair and regeneration [70]. The present study demonstrated that both 25(OH)-VD3 and 1,25(OH)_2_-VD3 markedly increased pulmonary levels of CAMP, reversing its reduction induced by fibrosis development. Moreover, administration of both of the VD3 metabolites restored CAMP expression to values comparable with those observed in healthy VD3-sufficient mice. These findings are particularly relevant because CAMP represents one of the best-characterised transcriptional targets of VDR. Activation of the VDR signalling pathway directly stimulates transcription of the cathelicidin gene, thereby enhancing antimicrobial defence, regulating inflammatory responses and supporting epithelial regeneration [67,70]. Consequently, the restoration of CAMP expression observed in the present study provides indirect evidence of effective activation of VD3-dependent transcriptional programmes within pulmonary tissue. Furthermore, the collected data suggest that 25(OH)-VD3 and 1,25(OH)_2_-VD3 attenuate HP-pulmonary fibrosis by simultaneously suppressing profibrotic signalling pathways and enhancing endogenous regenerative mechanisms.

The multivariate perspective provided by PCA corroborates the univariate findings across all analytical layers by integrating physiological, molecular, immunological, and histopathological variables into a unified representation of disease progression and treatment response. Such dimensionality-reduction approaches are particularly valuable in complex biological datasets because they capture coordinated changes across multiple variables rather than isolated effects, thereby providing a systems-level overview of therapeutic efficacy [71,72]. Across all analytical domains, PC1 consistently discriminated the SE-PA mice from the healthy controls, whereas the VD3-treated groups occupied intermediate positions, with the 1,25(OH)_2_-VD3-treated animals clustering reproducibly closer to the healthy phenotype than the 25(OH)-VD3-treated mice. This concordance across independent analytical platforms strengthens the conclusion that VD3-metabolites induce a coordinated, rather than compartment-specific, improvement in pulmonary function, inflammatory status, extracellular matrix remodelling, and molecular markers of fibrosis. Importantly, however, the PCA also demonstrates that treatment produced only a partial shift in the disease phenotype towards a healthy state rather than complete normalisation. Although the global disease signature represented by PC1 was consistently attenuated, the persistence of separation between the treated animals and healthy controls indicates that significant biological alterations remained unresolved. This finding deserves particular attention because it highlights both the therapeutic benefit and the biological limitations of the intervention. Such incomplete restoration is consistent with the concept that established pulmonary fibrosis involves multiple self-sustaining pathological programmes, some of which may become relatively independent of the initiating inflammatory stimulus, and, therefore, remain refractory to interventions targeting only selected regulatory pathways [21,25,73]. Of particular interest, CAMP emerged as a dimension largely independent of the fibrosis markers (hydroxyproline, FGF2, and TGFβ) in the ELISA-based PCA, supporting the interpretation that the restoration of CAMP expression by VD3 metabolites is driven predominantly by direct VDR-mediated transcriptional activation, rather than by indirect suppression of profibrotic signalling. The *Camp* gene contains a functional vitamin D response element (VDRE), making it one of the best-characterised direct transcriptional targets of activated VDR [74,75]. Consequently, the independent loading of CAMP on PC2 suggests that the activation of innate antimicrobial defence can occur even when the broader fibrotic programme remains only partially suppressed, emphasising the pleiotropic nature of VD3 metabolites. Similarly, the consistent separation of epithelial markers (*Cdh1*/E-cadherin and *Ocln*/occludin) onto PC2 in both RT-PCR and immunohistochemistry analyses indicates that epithelial integrity represents a biological dimension largely independent of the mesenchymal remodelling process. This observation mirrors current concepts of epithelial–mesenchymal transition (EMT), in which the loss of epithelial junction proteins and the acquisition of mesenchymal characteristics are related, but not perfectly coupled processes, regulated through overlapping yet distinct molecular networks [22,76]. In the present study, mesenchymal markers responded to VD3-treatment to a greater extent than epithelial markers, suggesting that fibrosis suppression does not necessarily translate into regeneration of the damaged alveolar epithelium. The inability of either 25(OH)-VD3 or 1,25(OH)_2_-VD3 to restore epithelial marker expression to control levels, therefore, represents one of the most important findings emerging from the multivariate analysis. The lack of changes in the expression of epithelial cell markers, despite the attenuation of fibrosis, implies that structural regeneration of the alveolar barrier may require additional mechanisms beyond the inhibition of profibrotic pathways, including the restoration of epithelial progenitor cell function, enhanced epithelial repair, or the stimulation of alveolar regeneration. This interpretation is consistent with growing evidence that failure of epithelial repair is a central driver of progressive pulmonary fibrosis, and that reversal of fibroblast activation alone may be insufficient to fully restore lung architecture [77,78]. Nevertheless, it should be emphasised once again that the lack of changes in the gene and protein expression of epithelial cell markers, instead of their enhancement noted in our earlier studies [18], may be explained by differences between preventive and therapeutic intervention paradigms.

Taken together, the PCA revealed that VD3 metabolites shift the overall disease profile toward a healthier phenotype across multiple biological levels, while simultaneously identifying the biological processes that remain resistant to treatment. The consistent intermediate positioning of the treated animals across all of the analytical layers suggests broad systemic efficacy, whereas the persistent segregation of epithelial markers identifies epithelial restoration as a remaining therapeutic gap. These findings reinforce the trend toward the superior efficacy of 1,25(OH)_2_-VD3 over 25(OH)-VD3, particularly for the endpoints where this advantage reached statistical significance in direct comparison, and indicate that future therapeutic strategies may benefit from combining VD3 signalling pathways with interventions specifically targeting epithelial regeneration and restoration of alveolar barrier integrity.

Despite the discovery of the great antifibrotic features of inhaled VD3 metabolites, the presented study has several limitations that should be considered when interpreting the results. In our opinion, the most important limitations include: the dose of VD3 metabolites administered, the sex of the animals, the age of the animals, the relatively small experimental group sizes, and the semi-quantitative nature of part of the collected data. (A) As mentioned previously, we based our strategy for preventing pulmonary fibrosis on VD3 metabolites delivered directly to the respiratory tract in doses intended to restore physiological calcitriol levels in the lungs of animals with dietary VD3 deficiency [18,19,38]. Although the doses of VD3 metabolites administered for 14 days to VD3-deficient mice with HP had successfully achieved this goal in our previous studies [19,38], the data collected in the present study revealed significant improvement in pulmonary calcitriol levels but not full recovery. We assume that this is the main reason why we observed the beneficial impact of inhaled VD3 metabolites on fibrosis development; however, this was without the complete neutralisation of the adverse changes induced by the antigen of *P. agglomerans*. This observation highlights the need to monitor calcitriol levels within the respiratory tract during therapy and to carefully adjust the therapeutic doses used, which could be the major challenge for the clinical implementation of therapeutic strategies based on VD3 metabolites. Therefore, dose–response studies that take into account both the stage of disease progression and pulmonary VD3 status are required to further optimise therapeutic dosing. (B) Although we consider the decision to conduct this pilot study exclusively in male mice to be justified and consistent with the 3Rs principle, which constitutes the ethical and legal framework for animal experimentation, this does not change the fact that, given the well-recognised sex-dependent differences in vitamin D3 metabolism [44], parallel studies in female animals would be highly desirable. Such studies would allow direct comparison with the findings presented in this manuscript. This issue is also important since sex-dependent differences in VD3 metabolism have to be considered when establishing the optimal therapeutic doses of VD3 metabolites. (C) The presented study was performed on 3-month-old mice, which represent adult individuals. Given the increasingly well-documented influence of ageing on the development of pulmonary fibrosis [79,80,81], it would also be justified to perform analogous studies in aged mice, while taking into account age-related alterations in VD3 metabolism. Similar to the factors discussed above, these changes are expected to play a crucial role in determining the optimal therapeutic dose. (D) The relatively small number of animals included in each experimental group also represents a limitation. Although statistically significant differences were observed for the majority of the analysed endpoints, studies involving larger cohorts are warranted to increase statistical power, improve the precision of effect estimates, and further validate the reproducibility of the findings. (E) In addition to quantitative analyses (including lung function assessment, characterization of immune cell subpopulations in lung tissue, and analysis of the expression of genes encoding transcription factors, as well as epithelial and mesenchymal cell markers, determination of fibrosis marker concentrations, and measurement of calcitriol levels in the lungs and serum), the expression of epithelial and mesenchymal cell markers, inflammatory changes, and signs of fibrosis were evaluated using semi-quantitative histopathological assessment and immunohistochemistry. Although these analyses were performed by an experienced histopathologist, who was blinded to the experimental group allocation throughout the entire assessment, they remain inherently subjective. Therefore, these findings should be interpreted with an appropriate degree of caution.

Finally, it is worth mentioning one additional aspect that has two sides. To the best of our knowledge, this is the first study to investigate the therapeutic potential of inhaled VD3 metabolites in which pharmacological treatment of pulmonary fibrosis was initiated during the acute phase of HP. There is a lack of comparable experimental data. Consequently, while the novelty of the experimental design represents a major strength of this study, the lack of independent reports from other research groups prevents direct comparison and discussion of the present observations. Further investigations performed in independent laboratories and across different experimental models will, therefore, be essential to confirm the reproducibility and translational relevance of these findings.

## 4. Materials and Methods

### 4.1. Reagents

Unless otherwise stated, the reagents were obtained from Sigma-Aldrich Co., LLC (Burlington, MA, USA),. The preparation of the *Pantoea agglomerans* antigen was described previously [15].

### 4.2. Research Design

The research was performed on three-month-old male C57BL/6 mice from the Mossakowski Medical Research Centre of the Polish Academy of Sciences in Warsaw, Poland (42 animals; mean weight 24.5 g). The research was conducted in the animal facility of the Institute of Rural Health in Lublin, Poland. After delivery, the animals were randomly assigned to research groups (6 mice/group). The animals were kept in separate polycarbonate cages under the following conditions: a natural light–dark cycle, temperature of 20–24 °C, air humidity of 45–65%, 15 air changes per hour, and unlimited access to fresh water and food. The VD3-sufficient mice (main control) received a diet with a standard cholecalciferol level (0.5 IU/g), while the VD3-deficient mice (other research groups, including control) received a diet with 10-times less cholecalciferol (0.05 IU/g), obtained from Altromin (Altromin Spezialfutter GmbH & Co. KG, Lage, Germany). After delivery, the animals underwent a week of acclimatisation, followed by a subsequent week of adaptation to the inhalation chambers and accessories (neck restrainers and nose-mouth silicone masks). The actual experiments began just after the indicated acclimatisation and adaptation procedures. VD3 statuses were confirmed by examination of pulmonary and serum levels of calcitriol [19,38]. The general health of the animals was monitored daily, as was body weight measurement. The mice were exposed to a saline extract of *P. aglomerans* at doses of 5 mg/mouse (SE-PA) or a 25(OH)-VD3 at a concentration of 100 pg/g or 1,25(OH)_2_-VD3 at a concentration of 5 pg/g. Doses of VD3 metabolites were chosen based on the results of our earlier studies [19,38], which showed that the restoration of the physiological pulmonary level of calcitriol in mice on a VD3-defficient diet (0.05 IU/g of cholecalciferol), with or without HP, require daily exposure to 100 pg/g of 25(OH)-VD3 or 5 pg/g of 1,25(OH)_2_-VD3 for at least 7 days. The 25(OH)-VD3 and 1,25(OH)_2_-VD3 were dissolved in 99.8% ethanol to concentrations of 100 ng/mL and 5 ng/mL, respectively. The stock solutions were divided into smaller portions and stored at −20 °C. The number of VD3-metabolites necessary to prepare working solutions was calculated based on the results of daily animal weight measurements. Working solutions obtained by appropriate dilution of the stock solutions in 10 mL of PBS were prepared immediately before inhalation. The freeze-dried antigen of *P. agglomerans* was also stored at −20 °C. Immediately before inhalation, 30 mg of antigen was dissolved in 10 mL of PBS and directly used for inhalation. The investigated agents were delivered directly to the mice’s respiratory tracts via nebulization, using the Buxco Inhalation Tower (Data Sciences International, St. Paul, MN, USA) under the following conditions: an airflow of 2.5 L/min, a pressure of −0.5 cm H_2_O, 20 °C, and an average nebulization rate of 351 μL/min.

**Figure 9:**
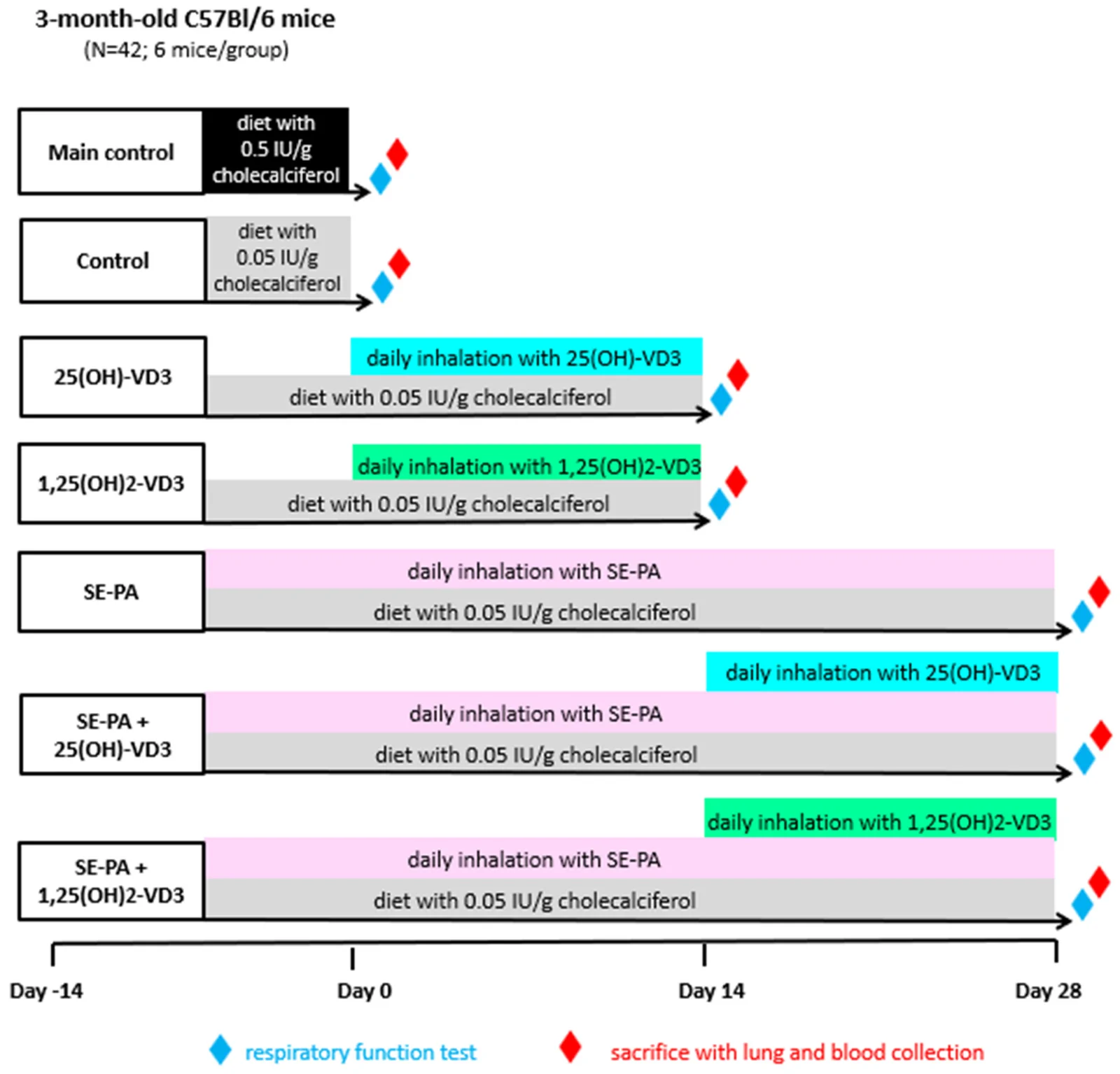
Research plan.

The final step of each experiment was whole-body plethysmography. The mice were placed in a non-invasive airway mechanics plethysmograph chamber (Data Sciences International, St. Paul, MN, USA). Respiratory parameters, including the frequency of breathing (F), minute volume (MV), accumulated volume (AV), time of inspiration (Ti), time of expiration (Te), mid-tidal expiratory flow (EF50), Total Cycle Time (TCT), and Inspiratory Duty Cycle (IDC), were determined using DSI FinePointe Software v 2.3.1.21 under the following conditions: an airflow of 0.6 L/min; a pressure of 0 cm H_2_O; and room temperature. Each experiment ended with the humane killing of the mice via cervical dislocation with spinal cord rupture, followed by dissection and collection of blood and lungs for additional studies. The graphical summary of the research design is presented in Figure 9, while the description of the investigated research groups is presented in Table 1. The research protocols were approved by the Local Ethics Committee for Animal Experimentation in Lublin, Poland (Resolution Nos. 28/2021, 25/2022, and 73/2022).

### 4.3. Evaluation of Calcitriol Concentration—ELISA

A description of the sample preparation for the determination of the calcitriol concentration has been presented previously [38]. The calcitriol serum level was determined by the Mouse 1,25-dihydroxyvitamin D3 (DVD/DHVD3) ELISA (Shanghai Coon Koon Biotech Co., Ltd., Shanghai, China), according to the instructions. The pulmonary level of calcitriol was determined by 1,25(OH)2 vitamin D Total ELISA (BioVendor R&D, Brno, Czech Republic), according to the instructions. The data collected from the lung tissue examination was normalised to the total protein concentration in the investigated homogenates, as described previously [38].

### 4.4. Evaluation of Immune Cells Composition—Flow Cytometry

A detailed description of lung tissue preparation for flow cytometry examination has been described previously [19]. Immune cells presented in lung homogenates were classified based on the following markers: dendritic cells (CD11b, CD103, and CD209), lymphocytes B (CD19 and B220), lymphocytes Tc (CD3 and CD8), lymphocytes Th (CD3 and CD4), lymphocytes Treg (CD4, CD25, and FoxP3), M1 macrophages (CD11b, CD86, and F4/80), M2 macrophages (CD11b, CD206, and F4/80), and neutrophils (CD11b and Ly-6G/Gr-1). Antibodies against CD206 and its isotype control were purchased from the Life Technologies Corporation (Carlsbad, CA, USA), while other antibodies and corresponding isotype controls were obtained from BD Biosciences (San Diego, CA, USA). A description of immune cell staining has been presented previously [19]. Stained cells were examined by using a BD Accuri C6 Plus flow cytometer with BD CSampler Plus software v. 1.0.34 (BD Biosciences, San Jose, CA, USA). The gating strategies for both surface and intracellular staining are presented in Figure S3. The representative gating plots for immune cell populations across all of the experimental groups were presented in Figure S4.

### 4.5. Assessment of Lung Tissue Injury Scores—Masson’s Trichrome Staining

Lung tissues were fixed in 4% buffered formalin. After dehydration, the lung samples were embedded in paraffin wax and then cut into small pieces and stained with Masson’s trichrome. The lungs were next evaluated under a MW50 light microscope (OPTA-TECH, Warsaw, Poland) by an experienced histopathologist for signs of inflammation and fibrosis. The histopathologist was blinded to the experimental group allocation throughout the entire assessment. Lung injury was graded using the 5-point Murray’s scale: 0 points = regular tissue; 1 point = slight injury to 25%; 2 points = moderate injury to 50%; 3 points = severe injury to 75%; and 4 points very severe injury around 100%. The lung injury scores were analysed twice, and the final visual scores were presented as the median of the investigated items. To visualise the changes in lung tissue morphology, micrographs were prepared using Capture V2.0 software (OPTATECH, Warsaw, Poland).

### 4.6. Visualisation of EMT-Marker Expression—Immunohistochemistry

A detailed description of the assessment of EMT protein expression in Formalin-Fixed Paraffin-Embedded (FFPE) tissue has been described previously [17]. EMT markers (N-cadherin, fibronectin, occludin, E-cadherin, vimentin, and α-SMA) were detected using specific antibodies obtained from abcam (Cambridge, UK), while antigen–antibody reactions were visualized using appropriate Deko Real EnVision/HRP (DAB+) detection systems for mouse and rabbit primary antibodies (Agilent Technologies, Santa Clara, CA, USA). Changes in the expression of EMT-markers were assessed under an MW50 light microscope (OPTA-TECH, Warsaw, Poland) using the 5-point scale: 0 points = no reaction/expression among target cells; 1 point = positive reaction in less than 25% of target cells; 2 points = positive reaction in 26–50% target cells; 3 points = positive reaction in 51–75% target cells; and 4 points = positive reaction in over 75% target cells. The protein expression was examined by an experienced histopathologist, who was blinded to the experimental group allocation throughout the entire assessment. Changes in the amount of EMT markers were recorded using an MW50 light microscope with Capture V2.0 software (OPTA-TECH, Warsaw, Poland).

### 4.7. Assessment of Gene Expression—Real-Time PCR

A detailed description of nucleic acids isolation, as well as preparation (reverse-transcription) for the gene expression assay, has been presented previously [18]. Real-Time PCR was performed using TaqMan Fast Universal PCR MasterMix and TaqMan Gene Expression Assays (Mm00725412_s1 for *Acta2*; Mm02619580_g1 for *Actb*; Mm01247357_m1 for *Cdh1*; Mm01162497_m1 for *Cdh2*; Mm01256744_m1 for *Fn1*; Mm00500912_m1 for *Ocln*; Mm00441533_g1 for *Snail1*; Mm00441531_m1 for *Snail2*; Mm01333430_m1 for *Vim*; Mm00495564_m1 for *Zeb1*; and Mm00497196_m1 for *Zeb2*) (Applied Biosystems, Foster City, CA, USA). The data were collected after 20 s at 95 °C incubation, followed by 40 cycles of 3 s incubation at 95 °C and 30 s at 60 °C, using a 7500 Fast Real-Time PCR System with SDS 1.4 Software (Applied Biosystems, Waltham, MA, USA). The relative gene expression levels were determined using relative advanced quantification and normalised to *Actb* expression.

### 4.8. Assessment of Protein Concentration—ELISA Method

A detailed description of the preparation of lung tissue homogenates for protein examination has been presented previously [38]. The number of proteins was determined using ELISA Kits dedicated to the following mouse proteins: CAMP (Cathelicidin) FGF2, hydroxyproline, and TGFβ (Cloud-Clone Corp., Katy, TX, USA).

### 4.9. Statistical Analysis

Group differences were assessed using the non-parametric Wilcoxon–Mann–Whitney test. *p*-values were adjusted for multiple testing with the Benjamini–Hochberg false discovery rate (FDR) procedure. An adjusted *p*-value below 0.05 was considered statistically significant. Effect sizes were determined via the Hodges–Lehmann estimator, which provides the median of all pairwise differences between observations in the compared groups. The statistical power of the Wilcoxon–Mann–Whitney test was estimated as the probability *p*(X > Y), using the shiehpow function from the wmwpow R package (version 0.1.3). Spearman’s rank correlation was used to assess relationships between variables. All computational work and data visualisation were performed in R version 4.5.0, using the following packages: ggplot2, patchwork, rstatix, wmwpow, broom, dplyr, and tidyr. The central line in the box plots represents the median, the box boundaries show the interquartile range (IQR), and the whiskers extend to the minimum and maximum observed values. Three distinct reference schemes were established for the visual comparisons: asterisks (*) indicate comparisons vs. the main control; tildes (~) indicate comparisons vs. the control; and carets (^) indicate comparisons between the treated and untreated mice with HP (SE-PA + 25(OH)-VD3 vs. SE-PA and SE-PA + 1,25(OH)2-VD3 vs. SE-PA). A single symbol denotes *p* < 0.05, two symbols *p* < 0.01, and three symbols *p* < 0.001.

### 4.10. Principal Component Analysis

PCA was performed separately for each analytical layer: whole-body plethysmography, RT-PCR gene expression, ELISA-based tissue repair factors, flow cytometry, and immunohistochemical assessment of EMT markers. Analytical layers containing fewer than three variables were excluded. Prior to analysis, the variables were centred and scaled to unit variance; only complete observations were retained. The PCA was computed using the prcomp function (R stats package v.4.5.0) and visualised as biplots using ggplot2 and patchwork.

## 5. Conclusions

The performed studies revealed that initiating treatment with VD3 metabolites administered by inhalation during the acute phase of HP significantly reduces the signs of fibrosis and the resulting respiratory disorders. The discovered therapeutic effects of inhaled 25(OH)-VD3 and 1,25(OH)_2_-VD3 involved the suppression of inflammatory cell recruitment, attenuation of profibrotic cytokine production, inhibition of EMT activation, and reduction in the extracellular matrix deposition. Among the tested compounds, 1,25(OH)_2_-VD3 demonstrated significantly superior efficacy to 25(OH)-VD3, specifically in suppressing pulmonary neutrophil and Tc lymphocyte infiltration and EMT-related gene expression (*Zeb2*, *Snail2*, and *Fn1*), while showing a consistent, but not yet statistically confirmed, numerical advantage for both the inflammatory and fibrosis score, hydroxyproline concentration, and respiratory function, as well as expression of the key EMT-inducer TGFβ. This pattern may result from bypassing local metabolic activation steps required for the conversion of 25(OH)-VD3 into the biologically active hormone. Moreover, a stronger effect of 1,25(OH)_2_-VD3 compared with 25(OH)-VD3 suggests that direct activation of VDR-dependent signalling pathways may represent a promising strategy for limiting pulmonary fibrosis associated with HP. The beneficial therapeutic effect of the investigated VD3 metabolites was associated with the elimination of disorders caused by the disease, and a cholecalciferol-restricted diet affected the amount of calcitriol. Since the tested interventions were not able to restore the physiological levels of calcitriol in lung tissue, this may explain the limited effectiveness of the therapy based on inhaled VD3 metabolites. Nevertheless, the obtained data encourage further investigation of local VD3-based interventions as a promising approach for HP. Future research should primarily focus on optimising therapeutic doses, taking into account age-, gender-, and disease stage-dependent changes in pulmonary calcitriol levels. To increase the chances of effective and safe HP therapy based on inhaled VD3 metabolites, it is crucial to identify and understand the molecular mechanisms responsible for their beneficial effects on the course of pulmonary fibrosis. Analysis should encompass key signalling pathways linking VD3 metabolism and EMT, such as TGF-β/Smad, NF-κB, and Wnt/β-catenin, as well as consider age- and gender-dependent changes in these responses. Accordingly, additional studies must be conducted in the same way, as well as additional animal models of pulmonary fibrosis, and after positive verification of the herein presented data, can be extended to clinical trials.

A graphical summary presenting changes in the mice with HP’s response to inhalation of 25(OH)-VD3 or 1,25(OH)_2_-VD3 is shown in Supplementary Figure S5.

## Figures and Tables

**Table 1:** Description of research groups.

Research Group (Mice Number)	Diet Content of VD3	SE-PA (5 mg/Mouse)	25(OH)-VD3 (100 pg/g)	1,25(OH)_2_-VD3 (5 pg/g)
**Main control** (n = 6)	0.5 IU/g	-	-	-
**Control** (n = 6)	0.05 IU/g	-	-	-
**25(OH)-VD3** (n = 6)	0.05 IU/g	-	30 min of daily inhalation for 14 consecutive days	-
**1,25(OH)2-VD3** (n = 6)	0.05 IU/g	-	-	30 min of daily inhalation for 14 consecutive days
**SE-PA** (n = 6)	0.05 IU/g	30 min of daily inhalation for 28 consecutive days	-	-
**SE-PA + 25(OH)-VD3** (n = 6)	0.05 IU/g	30 min of daily inhalation for 28 consecutive days	30 min of daily inhalation for 14 consecutive days, starting after 14 days of mice inhalation of SE-PA	-
**SE-PA + 1,25(OH)2-VD3** (n = 6)	0.05 IU/g	30 min of daily inhalation for 28 consecutive days	-	30 min of daily inhalation for 14 consecutive days, starting after 14 days of mice inhalation of SE-PA

## Data Availability

The original contributions presented in this study are included in the article/Supplementary Material. Further inquiries can be directed to the corresponding author.

## Supplementary Materials

The following supporting information can be downloaded at: https://www.mdpi.com/article/10.3390/molecules31162812/s1, Figure S1: Correlations between the concentrations of proteins involved in tissue repair and fibrosis score across all research groups; Figure S2: Correlations between calcitriol concentration and fibrosis score across all research groups; Figure S3: The gating strategies for both surface and intracellular staining; Figure S4: The representative gating plots for immune cell populations across all of the experimental groups; Figure S5: Graphical summary presenting changes in HP-mice response to inhalations with 25(OH)-VD3 or 1,25(OH)2-VD3; Table S1: Quantification of inflammatory and fibrosis in murine lung tissue in response to inhalation with antigen of *Pantoea agglomerans* and/or vitamin D3 metabolites; Table S2: Changes in lung function observed in VD3-deficient mice with HP treated with 25(OH)-VD3 or 1,25(OH)2-VD3; Table S3: Alterations in pulmonary composition of immune cells observed in VD3-deficient mice with HP treated with 25(OH)-VD3 or 1,25(OH)2-VD3; Table S4: Alterations in the gene expression of EMT markers observed in VD3-deficient HP-mice treated with 25(OH)-VD3 or 1,25(OH)2-VD3; Table S5: Alterations in the expression of EMT markers observed in VD3-deficient HP-mice treated with 25(OH)-VD3 or 1,25(OH)2-VD3; Table S6: Alterations in the expression of fibrosis markers and factors regulated tissue repair in VD3-deficient mice with HP in response to 25(OH)-VD3 or 1,25(OH)2-VD3; Table S7: Changes in serum and pulmonary levels of calcitriol in response to VD3-deficient mice with HP to treatment with 25(OH)-VD3 or 1,25(OH)2-VD3; Table S8: Direct pairwise comparison between SE-PA+25(OH)-VD3 and SE-PA+1,25(OH)2-VD3 treatment groups across all 40 parameters presented in Figure 1, Figure 2, Figure 3, Figure 4, Figure 5, Figure 6 and Figure 7.
