# Supplementary material for: The Therapeutic Efficacy of Inhaled Vitamin D3 Metabolites Initiated During the Acute Phase of Hypersensitivity Pneumonitis: An In Vivo Study
Source: Molecules. 2026 Aug 12;31(16):2812. doi: 10.3390/molecules31162812 (PMC13515577; doi:10.3390/molecules31162812)
Supplement: Supplementary file 1 [file molecules-31-02812-s001.zip › Figure S1.pdf]

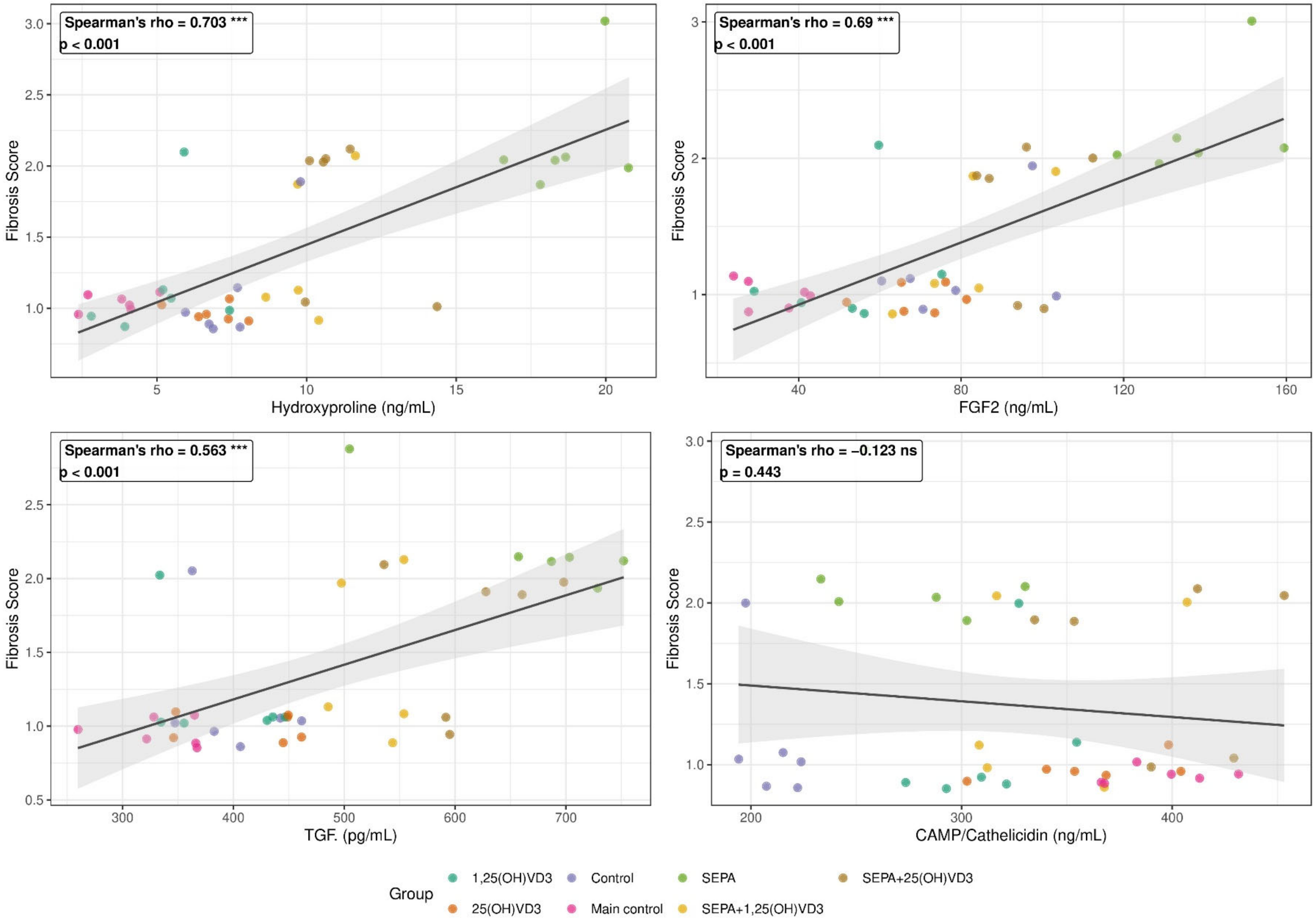

Figure S1. Correlations between the concentrations of proteins involved in tissue repair and fibrosis score across all research groups. Spearman's rank correlation was used to assess relationships between pulmonary concentrations of Hydroxyproline, FGF2, TGFβ, and CAMP determined by ELISA and fibrosis score assessed by histopathological examination. Each dot represents one animal; the regression line with 95% confidence interval is shown. Spearman's rho and corresponding p-values are indicated on each panel.
