# Supplementary material for: The Therapeutic Efficacy of Inhaled Vitamin D3 Metabolites Initiated During the Acute Phase of Hypersensitivity Pneumonitis: An In Vivo Study
Source: Molecules. 2026 Aug 12;31(16):2812. doi: 10.3390/molecules31162812 (PMC13515577; doi:10.3390/molecules31162812)
Supplement: Supplementary file 1 [file molecules-31-02812-s001.zip › Figure S2.pdf]

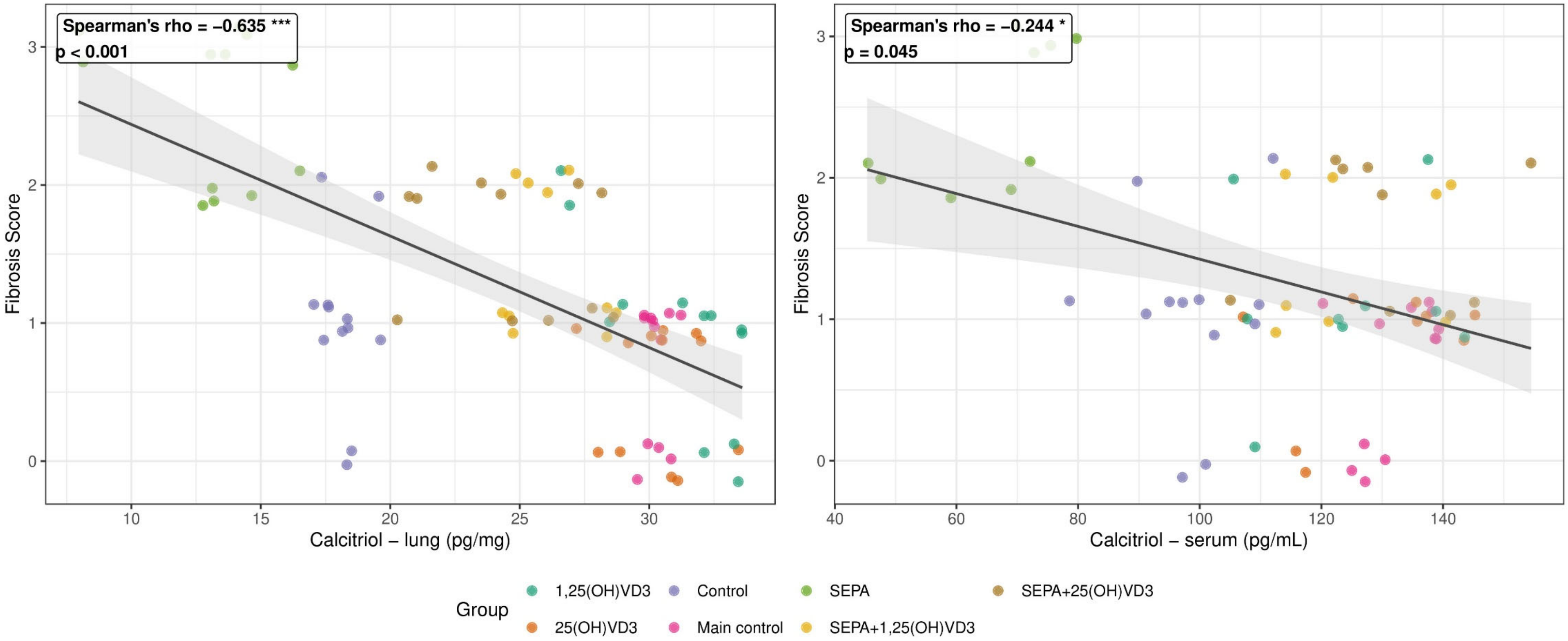

Figure S2. Correlations between calcitriol concentration and fibrosis score across all research groups. Spearman's rank correlation was used to assess relationships between serum and pulmonary levels of calcitriol determined by ELISA and fibrosis score assessed by histopathological examination. Each dot represents one animal; the regression line with 95% confidence interval is shown. Spearman's rho and corresponding p-values are indicated on each panel.
