# Supplementary material for: The Therapeutic Efficacy of Inhaled Vitamin D3 Metabolites Initiated During the Acute Phase of Hypersensitivity Pneumonitis: An In Vivo Study
Source: Molecules. 2026 Aug 12;31(16):2812. doi: 10.3390/molecules31162812 (PMC13515577; doi:10.3390/molecules31162812)

Figure S5. Graphical summary presenting changes in HP-mice response to inhalations with 25(OH)-VD3 or 1,25(OH)$_2$-VD3. The statistically significant increase in the investigated parameter value between the compared research groups was marked in green, while a decrease was marked in red. The lack of statistically significant change between the compared research groups was marked in grey.

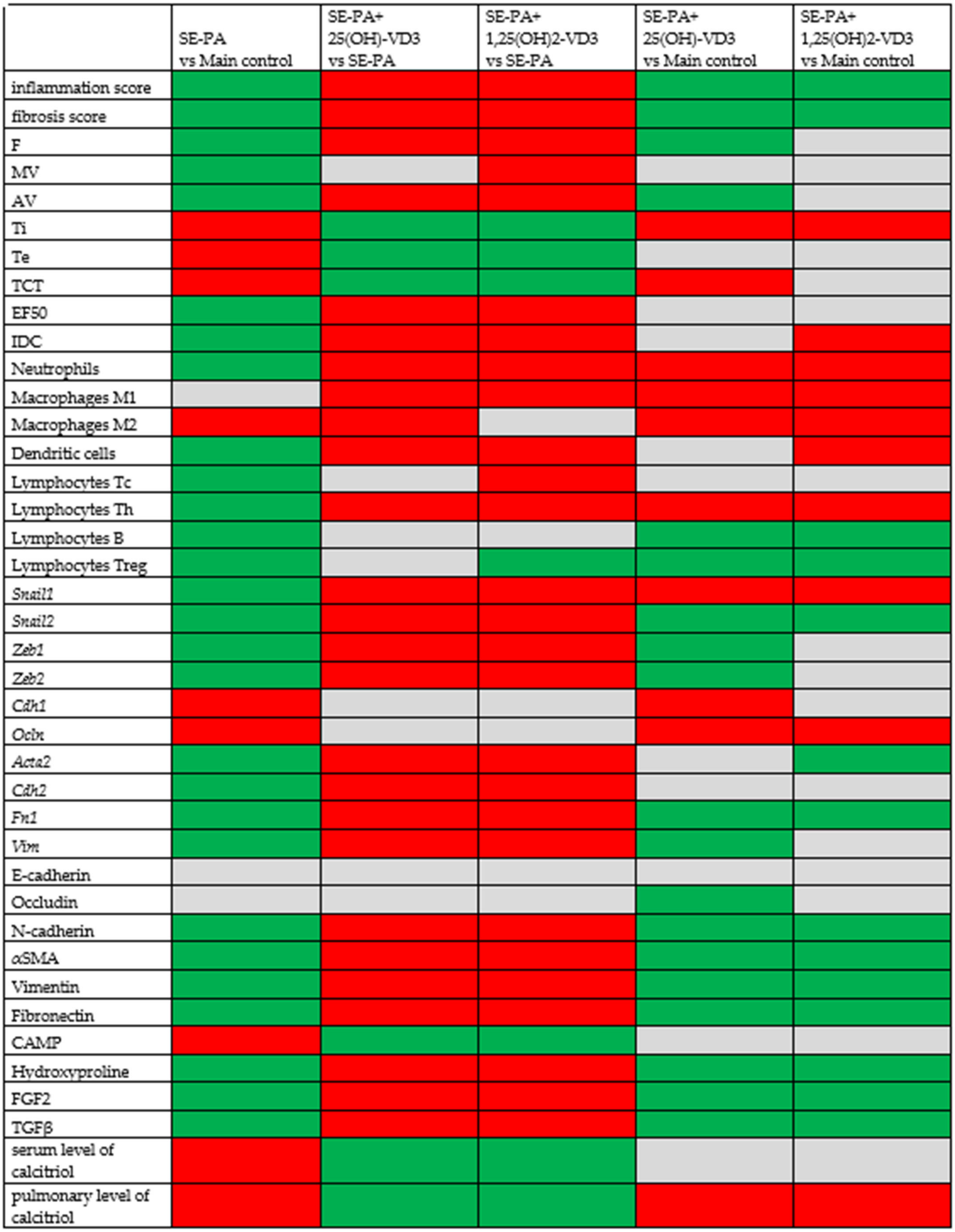

Supplement: Supplementary file 1 [file molecules-31-02812-s001.zip › Figure S5 new.pdf]
