# Supplementary material for: The Therapeutic Efficacy of Inhaled Vitamin D3 Metabolites Initiated During the Acute Phase of Hypersensitivity Pneumonitis: An In Vivo Study
Source: Molecules. 2026 Aug 12;31(16):2812. doi: 10.3390/molecules31162812 (PMC13515577; doi:10.3390/molecules31162812)
Supplement: Supplementary file 1 [file molecules-31-02812-s001.zip › Table S1 Histology.pdf]

Table S1. Quantification of inflammatory and fibrosis in murine lung tissue in response to inhalation with antigen of *Pantoea agglomerans* and/or vitamin D3 metabolites. Data for histologic scores are given as median of investigated items. Statistical significance was examined using the Wilcoxon-Mann-Whitney test with Benjamini-Hochberg correction. Statistical comparisons are presented for: * (vs. Main control); ~ (vs. Control); ^ (SE-PA+25(OH)-VD3 vs. SE-PA and SE-PA+1,25(OH)2-VD3 vs. SE-PA). The number of symbols correspond with the significance level: one ($p < 0.05$), two ($p < 0.01$), or three ($p < 0.001$).

	**Main control**	**Control**	**25-(OH)-VD3**	**1,25-(OH)$_2$-VD3**	**SE-PA**	**SE-PA + 25-(OH)-VD3**	**SE-PA + 1,25-(OH)$_2$-VD3**
**Inflammatory score**	0	0	0	0	~~~ *** 3	~~~ ^^^ *** 2	~~~ ^^^ *** 2
**Fibrosis score**	1	1	1	1	~~~ *** 3	~ ^^^ *** 2	^^^ ** 1
