# Supplementary material for: The Therapeutic Efficacy of Inhaled Vitamin D3 Metabolites Initiated During the Acute Phase of Hypersensitivity Pneumonitis: An In Vivo Study
Source: Molecules. 2026 Aug 12;31(16):2812. doi: 10.3390/molecules31162812 (PMC13515577; doi:10.3390/molecules31162812)
Supplement: Supplementary file 1 [file molecules-31-02812-s001.zip › Table S2 WBP.pdf]

Table S2. Changes in lung function observed in VD3-deficient mice with HP treated with 25(OH)-VD3 or 1,25(OH)$_2$-VD3. Respiratory parameters were investigated using the Whole Body Plethysmography. Data are presented as median.

	**Main control**	**Control**	**25-(OH)-VD3**	**1,25-(OH)$_2$-VD3**	**SE-PA**	**SE-PA + 25-(OH)-VD3**	**SE-PA + 1,25-(OH)$_2$-VD3**
**Frequency [breaths/min]**	261.87	291.75	303.82	288.49	399.64	327.26	287.06
**Minute Volume [mL/min]**	82.74	96.18	108.00	104.43	143.81	104.71	94.79
**Accumulated Volume [mL]**	434.24	471.58	603.95	519.75	807.20	573.93	486.44
**Time of inspiratory [s]**	0.10	0.09	0.09	0.09	0.07	0.08	0.08
**Time of expiratory [s]**	0.14	0.13	0.12	0.14	0.08	0.12	0.15
**Total Cycle Time [s]**	0.24	0.22	0.21	0.23	0.15	0.20	0.23
**EF50 [mL/s]**	3.46	3.99	4.28	4.52	6.15	4.55	4.08
**Inspiratory Duty Cycle [%]**	44.46	40.99	43.73	40.11	46.66	39.45	35.00
