# Supplementary material for: The Therapeutic Efficacy of Inhaled Vitamin D3 Metabolites Initiated During the Acute Phase of Hypersensitivity Pneumonitis: An In Vivo Study
Source: Molecules. 2026 Aug 12;31(16):2812. doi: 10.3390/molecules31162812 (PMC13515577; doi:10.3390/molecules31162812)
Supplement: Supplementary file 1 [file molecules-31-02812-s001.zip › Table S3 Flow cytometry.pdf]

Table S3. Alterations in pulmonary composition of immune cells observed in VD3-deficient mice with HP treated with 25(OH)-VD3 or 1,25(OH)$_2$-VD3. Flow cytometry data are presented as a median of percentage of all living immune cells

	**Main control**	**Control**	**25-(OH)-VD3**	**1,25-(OH)$_2$-VD3**	**SE-PA**	**SE-PA + 25-(OH)-VD3**	**SE-PA + 1,25-(OH)$_2$-VD3**
**Neutrophils CD11b+ Ly-6G+**	6.23	3.71	4.47	6.13	34.47	2.29	1.01
**Macrophages M1 CD11b+ F4/80+ CD86+**	7.93	0.53	2.13	2.13	10.09	0.81	0.58
**Macrophages M2 CD11b+ F4/80+ CD206+**	8.01	2.89	5.61	4.40	2.79	1.64	1.73
**Dendritic cells CD11b+ CD103+ CD209+**	6.05	2.90	2.24	2.32	9.20	4.65	3.75
**Lymphocytes Tc CD3+ CD8+**	6.64	4.24	2.15	3.93	9.36	8.02	6.31
**Lymphocytes Th CD3+ CD4+**	6.89	2.88	3.52	2.70	11.23	4.28	4.14
**Lymphocytes B B220+ CD19+**	0.36	0.19	0.05	0.30	0.87	1.03	0.75
**Lymphocytes Treg CD4+ CD25+ FOXP3+**	0.02	0.02	0.17	0.05	0.46	0.76	0.82
