# Supplementary material for: The Therapeutic Efficacy of Inhaled Vitamin D3 Metabolites Initiated During the Acute Phase of Hypersensitivity Pneumonitis: An In Vivo Study
Source: Molecules. 2026 Aug 12;31(16):2812. doi: 10.3390/molecules31162812 (PMC13515577; doi:10.3390/molecules31162812)
Supplement: Supplementary file 1 [file molecules-31-02812-s001.zip › Table S4 RT-PCR.pdf]

Table S4. Alterations in the gene expression of EMT markers observed in VD3-deficient HP-mice treated with 25(OH)-VD3 or 1,25(OH)$_2$-VD3. Gene expression was investigated in lung homogenates using real-time PCR methods. Data are presented as median.

	**Main control**	**Control**	**25-(OH)-VD3**	**1,25-(OH)$_2$-VD3**	**SE-PA**	**SE-PA + 25-(OH)-VD3**	**SE-PA + 1,25-(OH)$_2$-VD3**
Snail1	1.02	1.11	0.92	0.91	1.39	0.89	0.90
Snail2	1.03	1.39	1.01	0.96	2.08	1.42	1.32
Zeb1	0.99	1.34	1.00	0.97	2.10	1.24	1.01
Zeb2	1.02	1.35	1.01	0.88	1.86	1.26	1.07
Cdh1	1.03	1.00	0.92	0.97	0.77	0.74	0.76
Acta2	1.04	0.91	0.98	1.04	2.23	1.15	1.24
Cdh2	1.00	1.11	1.03	1.01	1.48	1.05	0.99
Ocln	1.00	0.96	0.91	0.93	0.42	0.53	0.50
Fn1	0.98	1.08	0.97	1.00	2.49	1.72	1.53
Vim	1.01	1.08	1.04	0.98	1.59	1.11	1.15
