# Supplementary material for: The Therapeutic Efficacy of Inhaled Vitamin D3 Metabolites Initiated During the Acute Phase of Hypersensitivity Pneumonitis: An In Vivo Study
Source: Molecules. 2026 Aug 12;31(16):2812. doi: 10.3390/molecules31162812 (PMC13515577; doi:10.3390/molecules31162812)
Supplement: Supplementary file 1 [file molecules-31-02812-s001.zip › Table S5 Immunohistochemistry.pdf]

Table S5. Alterations in the expression of EMT markers observed in VD3-deficient HP-mice treated with 25(OH)-VD3 or 1,25(OH)$_2$-VD3. Results obtained from visual quantification of protein expression on immunohistochemically stained lung tissue are presented as median.

	**Main control**	**Control**	**25-(OH)-VD3**	**1,25-(OH)$_2$-VD3**	**SE-PA**	**SE-PA + 25-(OH)-VD3**	**SE-PA + 1,25-(OH)$_2$-VD3**
**E-cadherin**	1	1	1	1	1	1	1
**N-cadherin**	1	1	1	1	2	1	1
**a-SMA**	0	0.5	1	1	2	1	1
**Occludin**	1	1	1	1	1	1	1
**Vimentin**	1	1	1	1	2	2	1.5
**Fibronectin**	1	1	1	1	2	2	2
