# Supplementary material for: The Therapeutic Efficacy of Inhaled Vitamin D3 Metabolites Initiated During the Acute Phase of Hypersensitivity Pneumonitis: An In Vivo Study
Source: Molecules. 2026 Aug 12;31(16):2812. doi: 10.3390/molecules31162812 (PMC13515577; doi:10.3390/molecules31162812)
Supplement: Supplementary file 1 [file molecules-31-02812-s001.zip › Table S6 ELISA.pdf]

Table S6. Alterations in the expression of fibrosis markers and factors regulated tissue repair in VD3-deficient mice with HP in response to 25(OH)-VD3 or 1,25(OH)$_2$-VD3. Protein concentration was determined in homogenates of lung tissue using ELISA Kits. Data are presented as medians.

	**Main control**	**Control**	**25-(OH)-VD3**	**1,25-(OH)$_2$-VD3**	**SE-PA**	**SE-PA + 25-(OH)-VD3**	**SE-PA + 1,25-(OH)$_2$-VD3**
**CAMP [ng/mL]**	391.51	211.27	361.18	315.33	271.28	400.95	316.82
**Hydroxyproline [ng/mL]**	3.92	7.40	6.92	5.43	18.43	10.69	9.76
**FGF2 [ng/mL]**	32.64	74.67	69.70	54.71	135.74	95.00	82.80
**TGFβ [pg/mL]**	346.53	394.52	446.85	392.92	695.23	584.90	543.85
