# Supplementary material for: The Therapeutic Efficacy of Inhaled Vitamin D3 Metabolites Initiated During the Acute Phase of Hypersensitivity Pneumonitis: An In Vivo Study
Source: Molecules. 2026 Aug 12;31(16):2812. doi: 10.3390/molecules31162812 (PMC13515577; doi:10.3390/molecules31162812)
Supplement: Supplementary file 1 [file molecules-31-02812-s001.zip › Table S7 ELISA calcitriol.pdf]

Table S7. Changes in serum and pulmonary levels of calcitriol in response to VD3-deficient mice with HP to treatment with 25(OH)-VD3 or 1,25(OH)$_2$-VD3. Calcitriol concentrations in serum and lung tissue homogenates were determined by dedicated ELISA. Data are presented as median.

	**Main control**	**Control**	**25-(OH)-VD3**	**1,25-(OH)$_2$-VD3**	**SE-PA**	**SE-PA + 25-(OH)-VD3**	**SE-PA + 1,25-(OH)$_2$-VD3**
**Serum level of calcitriol [pg/mL]**	132.71	98.64	135.54	123.52	69.86	130.08	121.19
**Pulmonary level of calcitriol [pg/mg]**	30.21	18.21	30.47	32.07	13.31	24.50	25.71
