# Supplementary material for: The Therapeutic Efficacy of Inhaled Vitamin D3 Metabolites Initiated During the Acute Phase of Hypersensitivity Pneumonitis: An In Vivo Study
Source: Molecules. 2026 Aug 12;31(16):2812. doi: 10.3390/molecules31162812 (PMC13515577; doi:10.3390/molecules31162812)
Supplement: Supplementary file 1 [file molecules-31-02812-s001.zip › Table S8 Statistic new.pdf]

Table S8. Direct pairwise comparison between SE-PA+25(OH)-VD3 and SE-PA+1,25(OH)2-VD3 treatment groups across all 40 parameters presented in Figures 1–7. Values recorded in mice treated with SE-PA+25(OH)-VD3 and SE-PA+1,25(OH)2-VD3 were compared directly using the Wilcoxon-Mann-Whitney test with Benjamini-Hochberg (BH) correction for multiple testing, consistent with the statistical framework described in Section 4.9. N — number of animals per treatment group; Median — median value recorded in each group; p-value — raw p-value from the Wilcoxon-Mann-Whitney test; p-value (BH-adjusted) — p-value corrected for multiple testing within the family of comparisons for each parameter; Significant — indicates whether the BH-adjusted p-value was below 0.05; Hodges-Lehmann difference — Hodges-Lehmann estimator of the median difference between groups (1,25(OH)2-VD3 minus 25(OH)-VD3). Parameters are grouped according to the corresponding main-text figure (Figure 1–7).

Figure	Parameter	Median 1 1,25(OH)2-VD3	Median 2 25(OH)-VD3	p-value	p-value (BH-adjusted)	Significant (adj. p < 0.05)	Hodges-Lehmann difference	N1/N2
Figure 1	Fibrosis score	1	2	0.425	0.469	No	0	10/12
Figure 1	Inflammatory score	2	2	0.496	0.496	No	0	10/12
Figure 2	Accumulated Volume (AV)	486,44	573,93	0.126	0.220	No	-93,134	5/6
Figure 2	Frequency of breathing (F)	287,06	327,26	0.082	0.133	No	-38,399	5/6
Figure 2	Inspiratory Duty Cycle (IDC)	35	39,45	0.126	0.198	No	-4,488	5/6
Figure 2	Mid-tidal Expiratory Flow (EF50)	4,08	4,55	0.177	0.252	No	-0,562	5/6
Figure 2	Minute Volume (MV)	94,79	104,71	0.177	0.252	No	-13,239	5/6
Figure 2	Time of expiratory (Te)	0,15	0,12	0.082	0.178	No	0,032	5/6
Figure 2	Time of inspiratory (Ti)	0,08	0,08	0.270	0.315	No	0,002	5/6
Figure 2	Total Cycle Time (TCT)	0,23	0,2	0.082	0.144	No	0,037	5/6
Figure 3	Dendritic cells	3,75	4,65	0.247	0.288	No	-1,4	5/6
Figure 3	Lymphocytes B	0,75	1,03	0.931	1.000	No	-0,055	5/6
Figure 3	Lymphocytes Tc	6,31	8,02	0.030	0.042	Yes	-1,705	5/6
Figure 3	Lymphocytes Th	4,14	4,28	0.314	0.314	No	-0,29	5/6
Figure 3	Lymphocytes Treg	0,82	0,76	0.116	0.129	No	0,18	5/6
Figure 3	Macrophages M1	0,58	0,8	0.329	0.384	No	-0,235	5/6
Figure 3	Macrophages M2	1,73	1,64	0.537	0.564	No	0,135	5/6

Figure 3	Neutrophils	1,01	2,29	0.004	0.008	Yes	-1,18	5/6
Figure 4	Acta2 (RT-PCR)	1,24	1,15	0.190	0.284	No	0,106	11/12
Figure 4	Cdh1 (RT-PCR)	0,76	0,74	0.449	0.591	No	0,065	11/12
Figure 4	Cdh2 (RT-PCR)	0,99	1,04	0.525	0.919	No	-0,059	11/12
Figure 4	Fn1 (RT-PCR)	1,53	1,72	0.037	0.052	No	-0,202	11/12
Figure 4	Ocln (RT-PCR)	0,5	0,53	1.000	1.000	No	0,001	11/12
Figure 4	Snail1 (RT-PCR)	0,9	0,89	0.975	0.977	No	0,013	11/12
Figure 4	Snail2 (RT-PCR)	1,32	1,42	0.007	0.010	Yes	-0,107	11/12
Figure 4	Vim (RT-PCR)	1,15	1,11	0.786	0.839	No	0,008	11/12
Figure 4	Zeb1 (RT-PCR)	1,01	1,24	0.196	0.257	No	-0,167	11/12
Figure 4	Zeb2 (RT-PCR)	1,07	1,26	< 0.001	< 0.001	Yes	-0,19	11/12
Figure 5	E-cadherin (IHC)	1	1	0.882	1.000	No	0	10/12
Figure 5	Fibronectin (IHC)	2	2	0.831	0.831	No	0	10/12
Figure 5	N-cadherin (IHC)	1	1	0.969	1.000	No	0	10/12
Figure 5	Occludin (IHC)	1	1	0.903	0.998	No	0	10/12
Figure 5	Vimentin (IHC)	1,5	2	0.731	0.853	No	0	10/12
Figure 5	α-SMA (IHC)	1	1	0.969	1.000	No	0	10/12
Figure 6	CAMP	316,82	400,95	0.082	0.115	No	-45,745	5/6
Figure 6	FGF2	82,8	95	0.126	0.155	No	-13,29	5/6
Figure 6	Hydroxyproline	9,76	10,69	0.177	0.186	No	-0,94	5/6
Figure 6	TGFβ	543,85	611,63	0.035	0.053	No	-95,504	5/6
Figure 7	Calcitriol - lung	25,71	24,5	0.159	0.186	No	1,445	10/12
Figure 7	Calcitriol - serum	121,19	130,08	0.190	0.333	No	-9,333	9/9
